# Updated Estimates of Patients With Oropharyngeal Cancer in the US

**DOI:** 10.1001/jamanetworkopen.2025.39258

**Published:** 2025-10-24

**Authors:** Caineng Cao, Anna Lee, Jung Julie Kang, Irini Yacoub, Kaveh Zakeri, Edward Christopher Dee, Nadeem Riaz, Achraf Shamseddine, Yao Yu, Jennifer Ma, Teeradon Treechairusame, Marc A. Cohen, Jennifer R. Cracchiolo, Richard J. Wong, Winston Wong, Lara A. Dunn, Eric J. Sherman, Nancy Y. Lee

**Affiliations:** 1 Department of Radiation Oncology, Zhejiang Cancer Hospital, Hangzhou Institute of Medicine, Chinese Academy of Sciences, Hangzhou, Zhejiang, China; 2 Department of Radiation Oncology, Memorial Sloan Kettering Cancer Center, New York, New York; 3 Department of Radiation Oncology, University of Texas MD Anderson Cancer Center, Houston; 4 Department of Therapeutic Radiology, Yale University School of Medicine, New Haven, Connecticut; 5 Department of Radiation Oncology, SUNY Downstate Health Sciences University, Brooklyn, New York; 6 Department of Surgery, Memorial Sloan Kettering Cancer Center, New York, New York; 7 Department of Medicine, Memorial Sloan Kettering Cancer Center, New York, New York

## Abstract

**Importance:**

Updated estimates of oropharyngeal cancer (OPC) in the US are needed.

**Objective:**

To calculate the most recent epidemiologic estimates of OPC in the US and provide projections for future trends up to 2040.

**Design, Setting, and Participants:**

This cross-sectional epidemiological analysis used data from the recent National Cancer Institute’s Surveillance, Epidemiology, and End Results (SEER) program. The SEER-22 (excluding Illinois and Massachusetts) database provided data for incidence, prevalence, survival, and initial treatment by OPC stage. Patients with OPC diagnosed according to *International Classification of Diseases for Oncology, Third Edition* morphology codes were included. The analysis was conducted from September to October 2024.

**Exposure:**

A diagnosis of OPC.

**Main Outcomes and Measures:**

The primary outcomes were annual age-adjusted OPC incidence per 100 000 persons, limited-duration prevalence, survival rate, and initial treatment. The incidence rate and the estimated annual percentage change for the most recent period were determined according to trend analysis from 2006 to 2021 and were used to estimate the incidence rate up to 2040.

**Results:**

There were 103 107 new OPC cases (40 051 patients [38.8%] aged ≥65 years; 82 820 male patients [80.3%]) recorded during 2006 to 2021. From 2006 to 2021, the incidence of OPC increased from 3.8 to 4.4 cases per 100 000 person-years. The projected incidence rates indicated a significant decrease for both female (1.1 cases per 100 000 person-years) and younger (aged <65 years, 2.0 cases per 100 000 person-years) patients in 2040. The 10-year limited-duration prevalence increased from 0.024% in 2012 to 0.033% in 2021. The 1-year period survival rate of OPC was 88.2% (95% CI, 87.7%-88.7%), the 3-year period survival rate was 76.5% (95% CI, 75.9%-77.1%), and the 5-year period survival rate was 69.2% (95% CI, 68.5%-69.9%). Of 7495 patients with OPC in 2021, 1621 (21.6%) were classified as receiving no treatment, 1647 (22.0%) received single treatment, and 4227 (56.4%) received multiple treatments initially. The distribution of treatments was similar from 2006 to 2021. Of 15 648 patients with localized stage disease, 7171 (45.8%) received no treatment. In 2021, more patients aged 65 years and older received no treatment compared with patients younger than 65 years (865 of 3525 patients [24.5%] vs 756 of 3970 patients [19.0%]).

**Conclusions and Relevance:**

In this cross-sectional study of OPC, the incidence of OPC in the US increased rapidly from 2006 to 2021 among male individuals, particularly among those aged 65 years and older. Although the distribution of treatment was similar through the assessed years, increased limited-duration prevalence and higher than previously reported survival were observed. A smaller proportion of patients with localized stage OPC were treated, especially among those aged 65 years and older, suggesting that further research is needed for optimal patient outcomes.

## Key Points

**Question:**

What do the latest US data reveal about oropharyngeal cancer (OPC) epidemiology?

**Findings:**

This cross-sectional study of 103 107 new OPC cases recorded from 2006 to 2021 found that OPC incidence increased overall, with the sharpest increase among older adults (ie, aged ≥65 years). OPC prevalence also increased during this period, with a 69.2% 5-year period survival rate, yet many older patients with localized disease received no treatment.

**Meaning:**

This study found that the incidence of OPC has increased among older patients, and although improved treatments may account for the increased prevalence and 5-year survival rate, localized disease in such patients appears to be undertreated, suggesting that further research is needed for optimal patient outcomes.

## Teaser

This cross-sectional study examines the most recent epidemiologic estimates of oropharyngeal cancer in the US and provides projections for future trends up to 2040.

## Introduction

Oropharyngeal cancer (OPC) incidence has increased among an expanding array of countries worldwide.^1^ In the US, OPC is the most common cancer associated with human papillomavirus (HPV).^2^ The US Food and Drug Administration has recommended vaccination for anogenital HPV infection and associated cancer prevention since 2006 for female individuals and since 2011 for male individuals.^3^ Unfortunately, vaccine completion rates are suboptimal. The pooled relative incidence estimates were 1.84 for HPV vaccine initiation and 1.50 for completion.^4^

According to the eighth edition of the American Joint Committee on Cancer staging system, OPC is classified as 2 distinct entities according to HPV status. For the favorable prognosis of HPV-associated OPC, deintensified treatments are being studied in clinical trials with various strategies under active investigation, including intensity-modulated proton therapy, transoral robotic surgery, and immunotherapy.^2,5,6^

The incidence trends for OPC from 2000 through 2017 in US have been reported previously.^7,8^ Given the use of HPV vaccines and advances in treatment, we hypothesized that limited-duration prevalence would increase. In addition, assessing current treatment approaches and projecting future OPC incidence is critical for guiding clinical research directions. This study aimed to provide updated epidemiological estimates of OPC in the US and project future trends using data from the National Cancer Institute’s Surveillance, Epidemiology, and End Results (SEER) program (2000-2021).

## Methods

### Data Sources

For this cross-sectional study, all data regarding demographic characteristics and cancer incidence were obtained from the SEER database spanning 2000 through 2021, derived from 22 registries across the US (excluding Illinois and Massachusetts),^9^ covering approximately 41.9% of the US population based on the 2020 census. This study was deemed to be exempt from full institutional review board review by Memorial Sloan Kettering Cancer Center, because of the retrospective nature of the study. The data were publicly available and deidentified, so informed consent was not needed. This study follows the Strengthening the Reporting of Observational Studies in Epidemiology (STROBE) reporting guidelines for cross-sectional studies. The analysis was conducted from September to October 2024.

### Study Population and Study End Points

Our analysis of incidence and initial treatment was based on patients who received a diagnosis of OPC starting in 2006 (the first year in which the HPV vaccine was approved by the US Food and Drug Administration). The analysis of prevalence and survival was based on the whole dataset of SEER-22 (excluding Illinois and Massachusetts). Cases of OPC were identified on the basis of their *International Classification of Diseases for Oncology, Third Edition *primary site codes as arising in the base of tongue (C019), lingual tonsil (C024), tonsils (C099, C111, and C142), uvula (C052), soft palate (C051), oropharynx lateral wall (C090, C091, C099, and C102), oropharynx anterior wall (C100 and C101), oropharynx posterior wall (C103).^10^ Incidence and limited-duration prevalence rates (5-year and 10-year) were calculated as the study end points.

### Statistical Analysis

To examine trends in incidence rates over time as annual percentage changes (APCs) and average APCs (AAPCs), we used the National Cancer Institute’s Joinpoint Regression Analysis program (version 5.0.2). Period survival is a method that enhances up-to-date monitoring of survival. In contrast to traditional cohort analysis of survival, period analysis derives long-term survival estimates exclusively from the survival experience of patients within some recent calendar period.^11^ First-course treatment (chemotherapy, radiation, and/or surgery) was reclassified; the details of treatments (eg, intensity-modulated proton therapy, transoral robotic surgery, or immunotherapy) were not included in the database. Data were shown in terms of no treatment, any treatment, single treatment, or multiple treatments (more than 1 treatment and chemoradiotherapy). Data were analyzed with the SEER*Stat statistical software version 8.4.3 (January 18, 2024), provided by SEER and SAS statistical software version 9.4 (SAS Institute).

In addition, we projected the future incidence rate of OPC including the overall rate and subgroup rates by gender and age. The incidence rate and the estimated APC for the most recent period were determined according to trend analysis from 2006 to 2021 and were used to estimate the incidence rate up to 2040. Specifically, if the APC for the most recent period was not statistically significant (ie, the 95% CI did not include the null value), the baseline value was set as the median of the incidence rates in the recent period, and it was assumed that the trend would remain unchanged. Conversely, if the APC was statistically significant, the baseline value was set as the incidence rate in 2021, and the incidence rate up to 2040 would be further estimated according to the corresponding APC.

## Results

There were 103 107 new OPC cases recorded during 2006 to 2021. Of these, 40 051 patients (38.8%) were aged 65 years and older and 82 820 (80.3%) were male. The most common subsites of OPC were the base of tongue (42 169 patients [40.9%]) and tonsil (43 615 patients [42.3%]). According to the SEER staging system, the stage distribution for all patients was 15 648 localized (15.2%), 67 248 regional (65.2%), 15 794 distant (15.3%), and 4417 (4.3%) unknown or unstaged. Details are shown in eTable 1 in Supplement 1.

### Incidence and Projection

**Figure 1.**
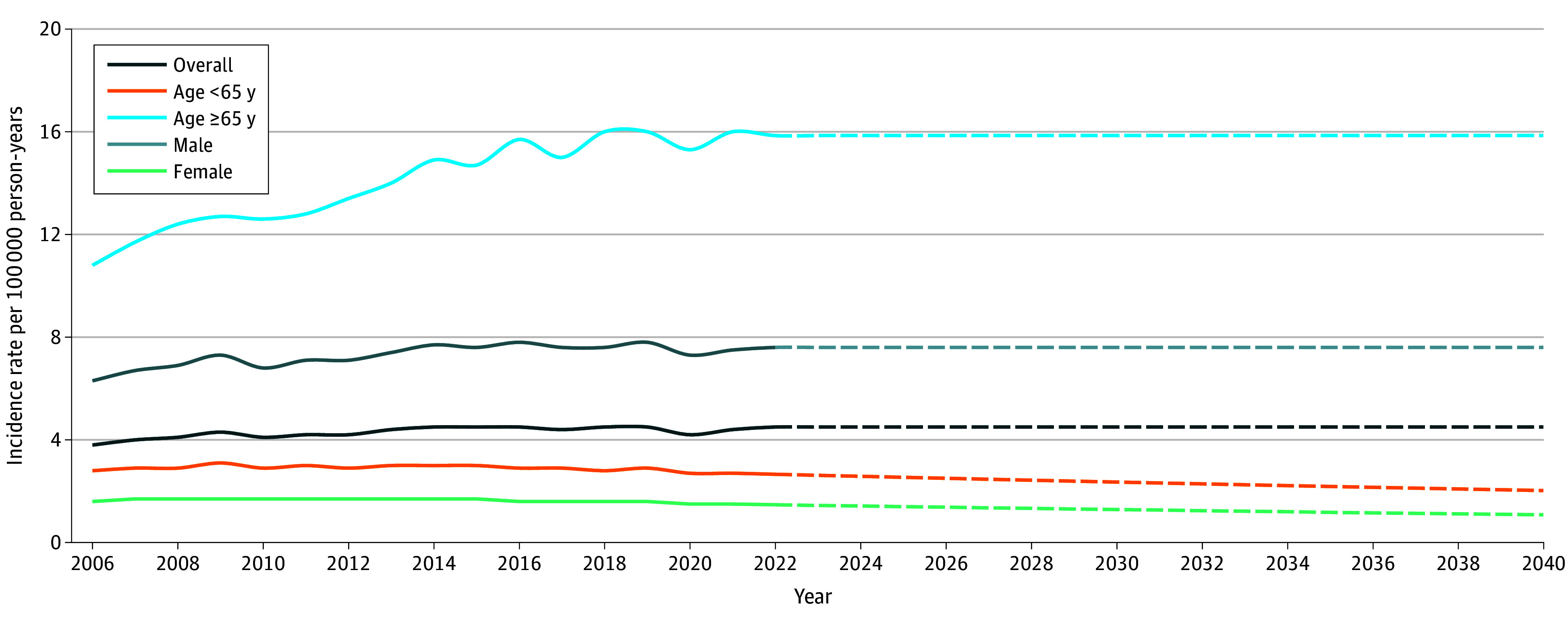
Incidence Trends and Projection of Oropharyngeal Cancer From 2006 to 2040 Solid lines denote actual incidence trends, and dashed lines denote projected incidence trends.

From 2006 to 2021, the incidence of OPC increased from 3.8 to 4.4 cases per 100 000 person-years (overall AAPC, 0.7%; 95% CI, 0.1% to 1.3%; *P* = .03). The incidence of OPC was consistently higher for older (≥65 years) than for younger (<65 years) patients. Among patients aged 65 years and older, incidence of OPC increased from 10.8 to 16.0 cases per 100 000 person-years (AAPC, 2.3%; 95% CI, 1.6% to 3.1%; *P* < .001). The incidence of OPC was markedly higher for male than for female patients, and the incidence increased from 6.3 to 7.5 cases per 100 000 person-years for male individuals (AAPC, 0.9%; 95% CI, 0.1% to 1.6%; *P* = .02). The incidence rates of OPC in the US from 2006 to 2021 by age and gender are presented in eTable 2 in Supplement 1. Figure 1 presents projections of future trends in OPC incidence rates in the US up to 2040, differentiated by age and gender. The projected incidence rates indicated a significant decrease for both female (1.1 cases per 100 000 person-years) and younger (aged <65 years, 2.0 cases per 100 000 person-years) patients in 2040.

The incidence of localized stage OPC was stable, and that for distant stage OPC decreased slightly from 0.7 to 0.6 cases per 100 000 person-years (AAPC for distant stage, −3.5%; 95% CI, −5.8% to −1.3%; *P* = .002), whereas regional stage increased from 2.3 to 3.1 cases per 100 000 person-years (AAPC for regional stage, 1.9%; 95% CI, 0.9% to 2.9%; *P* < .001). The absolute increase in regional stage incidence was observed among patients aged 65 years and older (5.8 to 10.6 cases per 100 000 person-years vs 1.7 to 2.0 cases per 100 000 person-years in the age <65 years cohort), although the relative increases in regional stage incidence were greater among patients aged 65 years and older. For distant stage OPC, the reduction in incidence was observed among patients aged 65 years and older (2.1 to 1.8 cases per 100 000 person-years; AAPC, −2.0%; 95% CI, −3.8% to −0.1%; *P* = .04). Age-specific incidence rates of OPC from 2006 to 2021 by age and stage are presented in eTable 3 in Supplement 1.

### Prevalence

**Figure 2.**
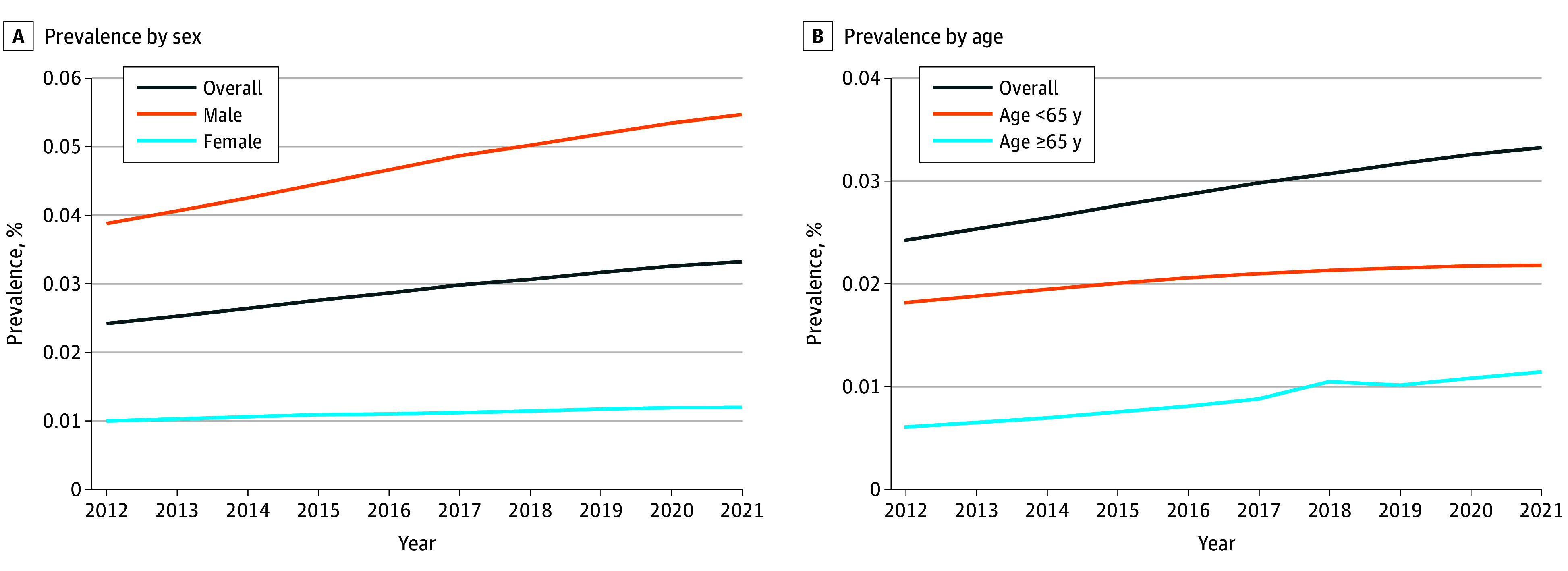
Limited Duration Prevalence of Oropharyngeal Cancer Graphs show prevalence by sex (A) and by age (B).

The 10-year limited-duration prevalence increased from 0.024% in 2012 to 0.033% in 2021. This trend was observed in both male and female patients over the same time frame; male patients had a higher prevalence than female patients in each year assessed (eg, in 2021, 0.055% for male patients vs 0.012% for female patients) (Figure 2A). A similar trend was observed in terms of 10-year limited-duration prevalence between age groups (≥65 years vs <65 years) (eTable 4 in Supplement 1 and Figure 2B).

### Survival

Estimates of survival based on SEER-22 (excluding Illinois and Massachusetts) are summarized in Table. The 1-year period survival rate for OPC was 88.2% (95% CI, 87.7%-88.7%), the 3-year period survival rate was 76.5% (95% CI, 75.9%-77.1%), and the 5-year period survival rate was 69.2% (95% CI, 68.5%-69.9%). Five-year period survival estimates were slightly lower for female vs male patients (67.0% [95% CI, 65.3%-68.6%] vs 69.7% [95% CI, 68.9%-70.4%]) and lower for patients aged 65 years and older vs those younger than 65 years (59.2% [95% CI, 58.0%-60.4%] vs 75.4% [95% CI, 74.5%-76.2%]). For patients with distant stage OPC, the overall 5-year survival estimate was 40.4% (95% CI, 38.3%-42.4%), and estimates were 34.7% (95% CI, 31.6%-37.8%) for those aged 65 years and older and 44.2% (95% CI, 41.4%-47.0%) for those younger than 65 years. When stratified by the rural-urban continuum code, patients in metropolitan areas exhibited marginally higher 5-year period survival rates than those in nonmetropolitan areas (70.0% [95% CI, 69.2%-70.7%] vs 64.7% [95% CI, 62.7%-66.6%]).

**Table.** Period Survival Rates of Patients With Oropharyngeal Cancer

Characteristic	Period survival rate, % (95% CI)				
	1 y	2 y	3 y	4 y	5 y
Overall	88.2 (87.7-88.7)	81.3 (80.7-81.9)	76.5 (75.9-77.1)	72.7 (72.1-73.4)	69.2 (68.5-69.9)
Age group, y					
<65	91.5 (91.0-92.0)	85.7 (85.0-86.4)	81.6 (80.8-82.3)	78.3 (77.5-79.1)	75.4 (74.5-76.2)
≥65	83.2 (82.3-84.1)	74.6 (73.5-75.6)	68.6 (67.5-69.7)	63.9 (62.8-65.1)	59.2 (58.0-60.4)
Gender					
Male	88.8 (88.3-89.3)	81.9 (81.3-82.5)	77.0 (76.3-77.7)	73.1 (72.4-73.9)	69.7 (68.9-70.4)
Female	85.5 (84.2-86.7)	78.6 (77.1-80.0)	74.2 (72.6-75.7)	70.9 (69.2-72.5)	67.0 (65.3-68.6)
Rural-urban continuum code					
Metropolitan	88.6 (88.1-89.1)	81.8 (81.1-82.4)	77.1 (76.4-77.8)	73.4 (72.7-74.1)	70.0 (69.2-70.7)
Nonmetropolitan	85.6 (84.2-87.0)	78.6 (76.9-80.2)	72.8 (71.0-74.5)	68.6 (66.7-70.4)	64.7 (62.7-66.6)
Summary stage					
Localized	92.7 (91.5-93.7)	87.2 (85.7-88.5)	83.5 (81.8-85.0)	80.0 (78.2-81.7)	76.6 (74.7-78.4)
Regional	90.7 (90.2-91.2)	84.6 (83.9-85.2)	79.9 (79.2-80.6)	76.5 (75.7-77.2)	73.0 (72.2-73.8)
Distant	67.2 (65.0-69.3)	54.6 (52.3-56.8)	48.0 (45.8-50.2)	43.5 (41.4-45.6)	40.4 (38.3-42.4)
Age group and summary stage					
Age <65 y					
Localized	95.9 (94.6-96.9)	92.8 (91.2-94.1)	90.2 (88.4-91.8)	88.2 (86.2-89.9)	85.9 (83.8-87.7)
Regional	93.6 (93.0-94.1)	88.4 (87.7-89.1)	84.4 (83.6-85.2)	81.4 (80.5-82.2)	78.5 (77.5-79.4)
Distant	70.5 (67.5-73.2)	57.6 (54.6-60.5)	51.3 (48.3-54.2)	46.9 (44.0-49.7)	44.2 (41.4-47.0)
Age ≥65 y					
Localized	88.6 (86.4-90.5)	79.9 (77.2-82.4)	74.7 (71.7-77.3)	69.3 (66.2-72.2)	64.4 (61.2-67.4)
Regional	86.1 (85.1-87.0)	78.2 (77.0-79.4)	72.3 (71.0-73.5)	68.1 (66.8-69.5)	63.5 (62.0-64.9)
Distant	63.3 (59.9-66.4)	50.8 (47.4-54.1)	43.8 (40.4-47.1)	39.0 (35.8-42.2)	34.7 (31.6-37.8)
Rural-urban continuum code and summary stage					
Metropolitan					
Localized	93.0 (91.7-94.1)	87.7 (86.1-89.2)	84.3 (82.5-85.9)	81.2 (79.3-82.9)	77.9 (75.9-79.7)
Regional	91.2 (90.6-91.7)	85.1 (84.4-85.7)	80.5 (79.8-81.3)	77.2 (76.4-78.0)	73.8 (72.9-74.6)
Distant	67.4 (65.0-69.6)	54.1 (51.7-56.5)	47.8 (45.4-50.1)	43.2 (40.9-45.5)	40.1 (37.9-42.3)
Nonmetropolitan					
Localized	91.0 (87.1-93.7)	83.7 (78.9-87.5)	78.2 (73.0-82.4)	73.0 (67.6-77.6)	68.8 (63.3-73.7)
Regional	87.9 (86.3-89.4)	81.5 (79.6-83.2)	75.9 (73.8-77.8)	72.0 (69.8-74.1)	68.1 (65.8-70.3)
Distant	66.1 (60.1-71.5)	57.3 (51.3-62.9)	49.6 (43.7-55.2)	45.6 (39.9-51.2)	42.0 (36.5-47.4)

### Initial Treatment

Of 7495 patients with OPC in 2021, 1621 (21.6%) were classified as receiving no treatment, 1647 (22.0%) received single treatment, and 4227 (56.4%) received multiple treatments initially. Of the patients receiving multiple treatments, 3584 patients (84.8%) received chemoradiotherapy. The distribution of treatments was similar from 2006 to 2021. In 2021, more patients aged 65 years and older received no treatment compared with patients younger than 65 years (865 of 3525 patients [24.5%] vs 756 of 3970 patients [19.0%]) and a lower proportion of patients aged 65 years and older received multiple treatments (1830 of 3525 patients [51.9%] vs 2397 of 3970 patients [60.4%]). Compared with male patients, female patients were more likely to receive no treatment (379 of 1351 female patients [28.1%] vs 1242 of 6144 male patients [20.2%]) and were less likely to undergo multiple treatments (638 of 1351 female patients [47.2%] vs 3589 of 6144 male patients [58.4%]). Similarly, nonmetropolitan areas demonstrated a higher proportion of untreated patients (268 of 1101 patients [24.3%] vs 1351 of 6389 patients [21.1%]) and fewer multitreatment interventions (587 of 1101 patients [53.3%] vs 3637 of 6389 patients [56.9%]) relative to metropolitan areas (eTable 5 in Supplement 1).

Of 15 648 patients with localized stage disease, 7171 (45.8%) received no treatment. Over the years, the rates of patients with localized stage disease undergoing surgery decreased from 25.0% (200 of 801 patients) in 2006 to 17.7% (203 of 1149 patients) in 2021. The proportions of patients with distant stage disease receiving no treatment ranged from 13.3% (130 of 975 patients) in 2009 to 21.6% (176 of 813 patients) in 2021, those receiving single treatment ranged from 21.4% (202 of 934 patients) in 2007 to 31.3% (237 of 756 patients) in 2018, and those receiving multiple treatments ranged from 47.1% (383 of 813 patients) in 2021 to 62.8% (612 of 975 patients) in 2010. The rates of patients receiving chemotherapy alone increased over the years from 16.5% (138 of 836 patients) in 2006 to 24.8% (202 of 813 patients) in 2021. A higher proportion of patients with distant stage disease aged 65 years and older received no treatment compared with those younger than 65 years in 2021 (107 of 403 patients [26.6%] vs 69 of 410 patients [16.8%]). In recent years, the proportions and distribution of single treatments for patients with distant stage disease were comparable between age groups, and fewer patients with distant stage disease aged 65 years and older received multiple treatments (eTable 6 in Supplement 1).

**Figure 3.**
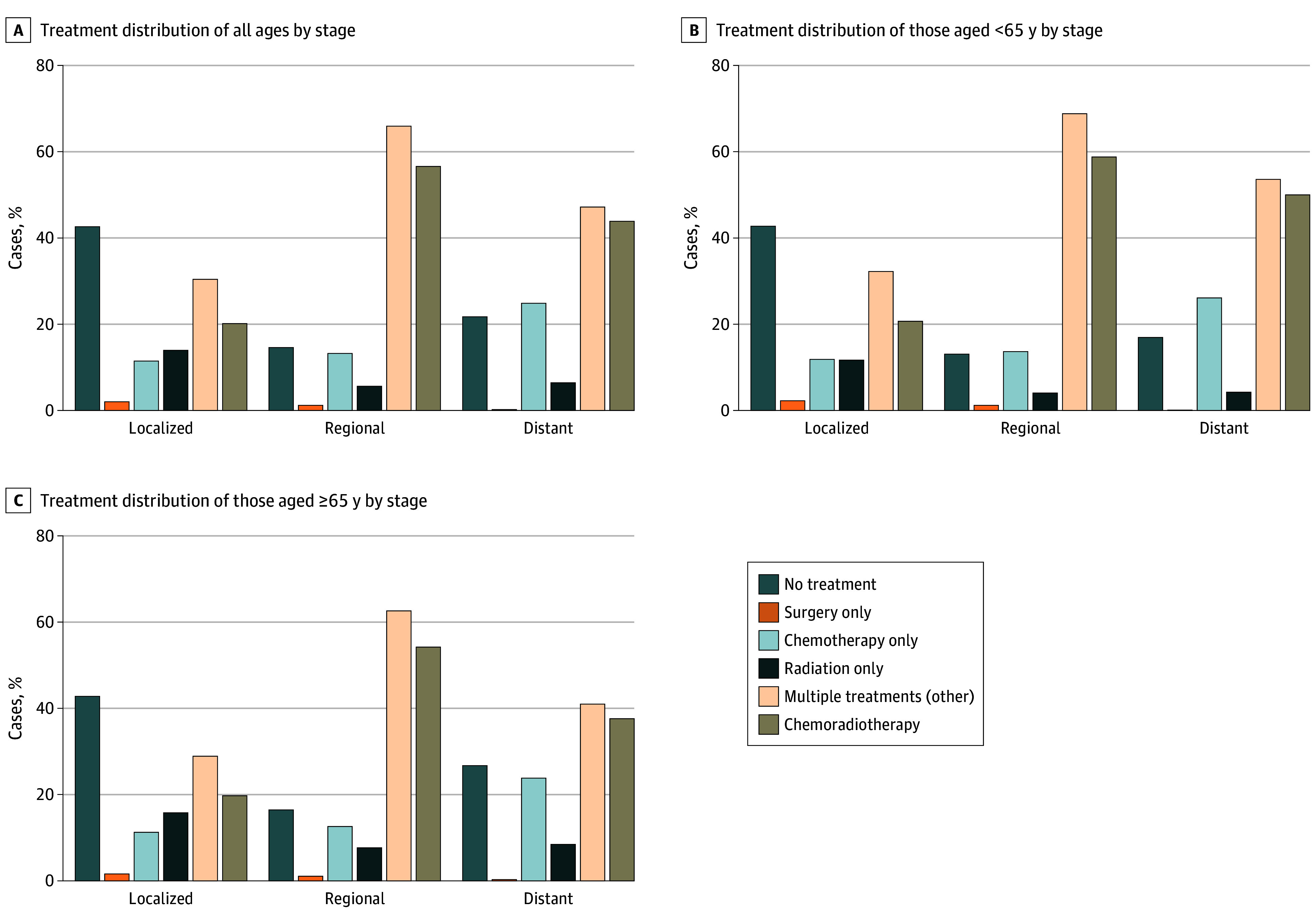
Distribution of Initial Treatment by Oropharyngeal Cancer Stage Graphs show treatment distribution for patients of all ages (A), for those younger than 65 years (B), and for those aged 65 years or older (C).

Treatment trends by age and stage in 2021 are shown in Figure 3. The proportions of patients receiving no treatment in the localized group were 42.6% (490 of 1149 patients), the proportion in the regional group was 14.5% (764 of 5286 patients), and that in the distant stage group was 21.6% (176 of 813 patients). Regardless of stage, a greater proportion of patients aged 65 years and older received no initial treatment compared with those younger than 65 years.

## Discussion

This cross-sectional study found that from 2006 to 2021, the incidence of OPC in the US increased rapidly among men, particularly among those aged 65 years and older. When analyzed by stage, the incidence of regional stage OPC increased, whereas that for distant stage OPC decreased. The incidence of localized stage OPC remained relatively stable. Although the distribution of treatments was similar through the assessed years, increased limited-duration prevalence and high survival rates were observed. A smaller proportion of patients with localized stage OPC, especially among those aged 65 years and older were treated.

OPC risk factors include HPV, smoking, and alcohol use.^1,7^ During the study period, the incidence of smoking-related and alcohol-related cancers such as hypopharyngeal cancer and lung cancer decreased across all age subgroups.^12,13^ It has been nearly 20 years since the approval of HPV vaccination.^3^ The incidences of cervical cancer and anal cancer decreased among young age groups in recent years.^14,15^ The median age of HPV-associated OPC was 60 years.^16^ Compared with the increased incidence among male patients and patients aged 65 years and older, the incidence of OPC slightly decreased among female patients and those younger than 65 years in recent years (eTable 2 in Supplement 1). The projected incidence rates also indicated a significant decrease for both female patients and younger patients.

According to the SEER-18 database, 3-year cause-specific survival rates were 60.8% for patients aged 65 years and older and 75.7% for patients aged 45 to 64 years.^8^ In that study, which included 2109 patients with OPC, the 5-year overall relative survival rate improved from 69% in 2006 to 2013 to 78% in 2014 to 2020. In addition, lower survival rates were observed in older patients and female patients.^17^ Similar survival outcomes and trends were observed in the present study. The increase in OPC among older patients was primarily associated with cancers in White male patients and tumors arising from the base of tongue and tonsil, consistent with HPV-associated cancers. The improvement in survival might be attributed to the increase in HPV-associated patients and the improvement in treatment.^16,18^

Consistent with the increase in survival and incidence, the limited-duration prevalence of OPC increased from 2012 to 2021. During the COVID-19 pandemic, global adult mortality rates markedly increased.^19^ No decrease was observed in limited-duration prevalence of OPC in 2020 and 2021, probably because of the increasing incidence of HPV-associated OPC.

Compared with HPV-negative OPC, HPV-associated OPCs are associated with a better radiosensitivity.^20^ Therefore, numerous radiochemotherapy de-escalation trials have been conducted since 2010, including optimal cisplatin administration or dosage, transoral robotic surgery–based protocols, and reduction of radiation doses and volumes. For locally advanced OPC, regardless of HPV status, concurrent cisplatin-based chemoradiotherapy was still one standard of care.^2^ For OPC, 65.2% of patients received a diagnosis of stage regional disease, and the distribution of treatments was similar from 2006 to 2021.

There has been increasing interest in primary surgical treatment such as transoral robotic surgery of patients with early T classification OPC.^21,22^ In a study of 8768 patients with category T1 to T2 OPC from the National Cancer Database, the proportions of patients undergoing primary surgery increased from 56% in 2004 to 82% in 2013.^21^ The rates of patients with localized stage disease undergoing surgery decreased probably because of the different sources of data and adoption of summary stage in the present article.

Despite availability of more effective treatments for OPC, a substantial proportion of patients (21.6% in 2021) may be undertreated. The classification of initial treatment was based on a study by Ganti et al.^13^ In that study, approximately 23% of all patients with non–small cell lung cancer were categorized as receiving no treatment in 2016, which is comparable to the results of the present study.^13^ The undertreated proportion was higher among patients aged 65 years and older vs those younger than 65 years (24.5% vs 19.0% in 2021). Patients with localized stage disease aged 65 years and older had the highest proportion with no treatment. These findings are not surprising, as previous study in resected non–small cell lung cancer have shown that limited numbers of older patients receive adjuvant chemotherapy, even though adjuvant chemotherapy is recommended.^23^ In patients with non–small cell lung cancer in the US, 38.3% of patients with stage IV disease aged 65 years and older received no treatment.^13^ Compared with patients with other cancers, patients with OPC are likely to be at higher risk of financial toxicity.^24^ Other reasons for not treating older patients with OPC may include comorbidities, balancing preferences for length of life with its quality, low Karnofsky Index, and older age.^25^ Undertreatment of female patients was another noteworthy trend observed, with 28.1% of female patients vs 20.2% of male patients receiving no treatment in 2021. Gender disparities in enrollment of women on clinical trials and their undertreatment have been reported, but root causes are poorly understood.^26^ Since the SEER-22 (excluding Illinois and Massachusetts) database only includes first-course radiation, chemotherapy, and surgery, some patients classified as no treatment may have received other treatments (eg, immunotherapy).

### Limitations

This study has limitations that should be mentioned. Aside from the retrospective nature, the SEER-22 (excluding Illinois and Massachusetts) database does not include the HPV status to distinguish between HPV-positive and HPV-negative OPC. A further caveat of this study is that the treatment and staging systems are not classified as typically done in the clinic. Furthermore, treatment factors, such as radiation therapy techniques, chemotherapy regimens, and surgery detail, are unavailable and may have confounded the results. Nevertheless, our report is noteworthy because it provides an important and comprehensive update of epidemiologic information for OPC.

## Conclusions

In this cross-sectional study of OPC, between 2006 and 2021, the incidence of OPC in the US increased rapidly among male patients, particularly among those aged 65 years and older. Although the distribution of treatment was similar through the assessed years, increased limited-duration prevalence and higher than previously reported survival were observed.

## Notes

Supplement 1. eTable 1. Baseline characteristics of patients diagnosed with oropharyngeal cancer from 2006 to 2021 in 22 SEER (excluding IL and MA) registries eTable 2. Incidence of oropharyngeal cancer in the US, 2006-2021 eTable 3. Incidence of oropharyngeal cancer in the US by stage (overall and age stratified), 2006-2021 eTable 4. 10-Year prevalence of oropharyngeal cancer in the US eTable 5. Initial treatment for oropharyngeal cancer in the US eTable 6. Initial treatment by stage, age and rural-urban continuum code

Supplement 2. Data Sharing Statement
