# Supplementary material for: Updated Estimates of Patients With Oropharyngeal Cancer in the US
Source: JAMA Netw Open. 2025 Oct 24;8(10):e2539258. doi: 10.1001/jamanetworkopen.2025.39258 (PMC12552931; doi:10.1001/jamanetworkopen.2025.39258)
Supplement: Supplement 1. — eTable 1. Baseline characteristics of patients diagnosed with oropharyngeal cancer from 2006 to 2021 in 22 SEER (excluding IL and MA) registries eTable 2. Incidence of oropharyngeal cancer in the US, 2006-2021 eTable 3. Incidence of oropharyngeal cancer in the US by stage (overall and age stratified), 2006-2021 eTable 4. 10-Year prevalence of oropharyngeal cancer in the US eTable 5. Initial treatment for oropharyngeal cancer in the US eTable 6. Initial treatment by stage, age and rural-urban continuum code [file jamanetwopen-e2539258-s001.pdf]

# Supplemental Online Content

Cao C, Lee A, Kang JJ, et al. Updated estimates of patients with oropharyngeal cancer in the US. *JAMA Netw Open*. 2025;8(10):e2539258.
doi:10.1001/jamanetworkopen.2025.39258

**eTable 1.** Baseline characteristics of patients diagnosed with oropharyngeal cancer from 2006 to 2021 in 22 SEER (excluding IL and MA) registries
**eTable 2.** Incidence of oropharyngeal cancer in the US, 2006-2021
**eTable 3.** Incidence of oropharyngeal cancer in the US by stage (overall and age stratified), 2006-2021
**eTable 4.** 10-Year prevalence of oropharyngeal cancer in the US
**eTable 5.** Initial treatment for oropharyngeal cancer in the US
**eTable 6.** Initial treatment by stage, age and rural-urban continuum code

This supplemental material has been provided by the authors to give readers additional information about their work.

© 2025 Cao C et al. *JAMA Network Open*.

**eTable 1. Baseline characteristics of patients diagnosed with oropharyngeal cancer from 2006 to 2021 in 22 SEER (excluding IL and MA) registries.**

| | | <65 years old (n=63,056) (%) | ≥65 years old (n=40,051) (%) | Total (n=103,107) | P-value |
|---|---|---|---|---|---|
| Year of diagnosis | | | | | < .001 |
| | 2006-2009 | 14,318 (22.7) | 6,886 (17.2) | 21204 (20.6) | |
| | 2010-2012 | 11,694 (18.5) | 6,170 (15.4) | 17864 (17.3) | |
| | 2013-2015 | 12,577 (19.9) | 7,751 (19.4) | 20328 (19.7) | |
| | 2016-2018 | 12,421 (19.7) | 9,110 (22.7) | 21531 (20.9) | |
| | 2019-2021 | 12,046 (19.1) | 10,134 (25.3) | 22180 (21.5) | |
| Gender | | | | | < .001 |
| | Male | 51,959 (82.4) | 30,861 (77.1) | 82820 (80.3) | |
| | Female | 11,097 (17.6) | 9,190 (22.9) | 20287 (19.7) | |
| Rural-urban continuum code | | | | | .06 |
| | Metropolitan | 54,534 (86.5) | 34,544 (86.3) | 89078 (86.4) | |
| | Nonmetropolitan | 8,471 (13.4) | 5,488 (13.7) | 13959 (13.5) | |
| | Unknown | 51 (0.1) | 19 (0.1) | 70 (0.1) | |
| Oropharynx subsites | | | | | < .001 |
| | Base of tongue | 23,268(36.9) | 18,901(47.2) | 42169 (40.9) | |
| | Tonsil | 29,576 (46.9) | 14,039 (35.1) | 43615 (42.3) | |

© 2025 Cao C et al. *JAMA Network Open*.

| | | | | | |
|---|---|---|---|---|---|
| | Soft palate | 2,235 (3.5) | 1,990 (5.0) | 4225 (4.1) | |
| | Other oropharynx | 7,977 (12.7) | 5,121 (12.8) | 13098 (12.7) | |
| Summary stage | | | | | < .001 |
| | Localized | 8,401 (13.3) | 7,247 (18.1) | 15648 (15.2) | |
| | Regional | 42,737 (67.8) | 24,511 (61.2) | 67248 (65.2) | |
| | Distant | 9,512 (15.1) | 6,282 (15.7) | 15794 (15.3) | |
| | Unknown/unstaged | 2,406 (3.8) | 2,011* (5.0) | 4417 (4.3) | |

*including in situ in 1.

© 2025 Cao C et al. *JAMA Network Open*.

**eTable 2. Incidence of oropharyngeal cancer in the US, 2006-2021.**

| | Incidence | N | SEER Population |
|---|---|---|---|
| Overall (2006-2021) | 4.3 | 103,107 | 2,117,681,515 |
| $APC_{overall}$ (2006-2015)=1.6%(0.9% to 2.4%) P < .001 | | | |
| $APC_{overall}$ (2015-2021)=-0.7%(-2.0% to 0.7%) P = .28 | | | |
| $AAPC_{overall}$=0.7%; 95% CI, 0.1% to 1.3%; P = .03 | | | |
| 2006 | 3.8 | 4,754 | 123,064,363 |
| 2007 | 4.0 | 5,167 | 124,343,542 |
| 2008 | 4.1 | 5,446 | 125,772,118 |
| 2009 | 4.3 | 5,837 | 127,188,118 |
| 2010 | 4.1 | 5,688 | 128,530,337 |
| 2011 | 4.2 | 6,017 | 129,847,860 |
| 2012 | 4.2 | 6,159 | 131,125,146 |
| 2013 | 4.4 | 6,504 | 132,308,152 |
| 2014 | 4.5 | 6,877 | 133,582,353 |
| 2015 | 4.5 | 6,947 | 134,860,706 |
| 2016 | 4.5 | 7,148 | 136,028,149 |
| 2017 | 4.4 | 7,108 | 137,008,306 |
| 2018 | 4.5 | 7,275 | 137,794,939 |
| 2019 | 4.5 | 7,523 | 138,472,337 |
| 2020 | 4.2 | 7,162 | 138,936,824 |
| 2021 | 4.4 | 7,495 | 138,818,265 |

© 2025 Cao C et al. *JAMA Network Open*.

| | Incidence | N | SEER Population |
|---|---|---|---|
| Age <65y (2006-2021) | | | |
| $APC_{age<65y}$(2006-2014)=0.6%(-0.3% to 1.5%) P = .15 | | | |
| $APC_{age<65y}$(2014-2021)=-1.5%(-2.6% to -0.5%) P = .009 | | | |
| $AAPC_{age<65y}$=-0.4%; 95% CI, -1.0% to 0.2%; P = .21 | | | |
| 2006 | 2.8 | 3,241 | 109,064,060 |
| 2007 | 2.9 | 3,506 | 110,084,040 |
| 2008 | 2.9 | 3,634 | 111,140,930 |
| 2009 | 3.1 | 3,937 | 112,205,761 |
| 2010 | 2.9 | 3,754 | 113,197,288 |
| 2011 | 3.0 | 3,992 | 114,159,908 |
| 2012 | 2.9 | 3,948 | 114,763,599 |
| 2013 | 3.0 | 4,117 | 115,375,732 |
| 2014 | 3.0 | 4,211 | 116,068,236 |
| 2015 | 3.0 | 4,249 | 116,765,266 |
| 2016 | 2.9 | 4,181 | 117,360,675 |
| 2017 | 2.9 | 4,180 | 117,779,934 |
| 2018 | 2.8 | 4,060 | 117,982,336 |
| 2019 | 2.9 | 4,198 | 118,057,077 |
| 2020 | 2.7 | 3,878 | 117,938,549 |
| 2021 | 2.7 | 3,970 | 117,273,851 |

© 2025 Cao C et al. *JAMA Network Open*.

| | Incidence | N | SEER Population |
|---|---|---|---|
| Age ≥ 65y (2006-2021) | | | |
| $APC_{age>65y}$(2006-2016)=3.3%(2.6% to 4.0%) P < .001 | | | |
| $APC_{age>65y}$(2016-2021)=0.5%(-1.5% to 2.5%) P = .58 | | | |
| $AAPC_{age>65y}$=2.3%; 95% CI, 1.6% to 3.1%; P < .001 | | | |
| 2006 | 10.8 | 1,513 | 14,000,303 |
| 2007 | 11.7 | 1,661 | 14,259,502 |
| 2008 | 12.4 | 1,812 | 14,631,188 |
| 2009 | 12.7 | 1,900 | 14,982,357 |
| 2010 | 12.6 | 1,934 | 15,333,049 |
| 2011 | 12.8 | 2,025 | 15,687,952 |
| 2012 | 13.4 | 2,211 | 16,361,547 |
| 2013 | 14.0 | 2,387 | 16,932,420 |
| 2014 | 14.9 | 2,666 | 17,514,117 |
| 2015 | 14.7 | 2,698 | 18,095,440 |
| 2016 | 15.7 | 2,967 | 18,667,474 |
| 2017 | 15.0 | 2,928 | 19,228,372 |
| 2018 | 16.0 | 3,215 | 19,812,603 |
| 2019 | 16.0 | 3,325 | 20,415,260 |
| 2020 | 15.3 | 3,284 | 20,998,275 |
| 2021 | 16.0 | 3,525 | 21,544,414 |

© 2025 Cao C et al. *JAMA Network Open*.

| | Incidence | N | SEER Population |
|---|---|---|---|
| Male (2006-2021) | | | |
| $APC_{male}$(2006-2016)=1.8%(1.1% to 2.5%) P < .001 | | | |
| $APC_{male}$(2016-2021)=-0.9%(-2.9% to 1.1%) P = .32 | | | |
| $AAPC_{male}$=0.9%; 95% CI, 0.1% to 1.6%; P = .02 | | | |
| 2006 | 6.3 | 3,694 | 60,649,507 |
| 2007 | 6.7 | 4,049 | 61,283,907 |
| 2008 | 6.9 | 4,289 | 61,996,457 |
| 2009 | 7.3 | 4,639 | 62,696,794 |
| 2010 | 6.8 | 4,440 | 63,365,159 |
| 2011 | 7.1 | 4,759 | 64,068,816 |
| 2012 | 7.1 | 4,892 | 64,770,449 |
| 2013 | 7.4 | 5,236 | 65,417,108 |
| 2014 | 7.7 | 5,537 | 66,112,921 |
| 2015 | 7.6 | 5,624 | 66,816,055 |
| 2016 | 7.8 | 5,827 | 67,459,552 |
| 2017 | 7.6 | 5,782 | 68,008,352 |
| 2018 | 7.6 | 5,906 | 68,454,165 |
| 2019 | 7.8 | 6,145 | 68,845,563 |
| 2020 | 7.3 | 5,857 | 69,117,849 |
| 2021 | 7.5 | 6,144 | 69,029,255 |

© 2025 Cao C et al. *JAMA Network Open*.

| | Incidence | N | SEER Population |
|---|---|---|---|
| Female (2006-2021) | | | |
| $APC_{female}$(2006-2013)=0.6%(-0.2% to 1.4%) P = .15 | | | |
| $APC_{female}$(2013-2021)=-1.7%(-2.3% to -1.0%) P < 0.001 | | | |
| $AAPC_{female}$=-0.6%; 95% CI, -1.1% to -0.2%; P = .007 | | | |
| 2006 | 1.6 | 1,060 | 62,414,856 |
| 2007 | 1.7 | 1,118 | 63,059,635 |
| 2008 | 1.7 | 1,157 | 63,775,661 |
| 2009 | 1.7 | 1,198 | 64,491,324 |
| 2010 | 1.7 | 1,248 | 65,165,178 |
| 2011 | 1.7 | 1,258 | 65,779,044 |
| 2012 | 1.7 | 1,267 | 66,354,697 |
| 2013 | 1.7 | 1,268 | 66,891,044 |
| 2014 | 1.7 | 1,340 | 67,469,432 |
| 2015 | 1.7 | 1,323 | 68,044,651 |
| 2016 | 1.6 | 1,321 | 68,568,597 |
| 2017 | 1.6 | 1,326 | 68,999,954 |
| 2018 | 1.6 | 1,369 | 69,340,774 |
| 2019 | 1.6 | 1,378 | 69,626,774 |
| 2020 | 1.5 | 1,305 | 69,818,975 |
| 2021 | 1.5 | 1,351 | 69,789,010 |

Incidence per 100,000 persons, age-adjusted to the 2000 US standard population (19 age groups - Census P25-1130). US, United States; y, years; APC, annual percentage change; AAPC, average annual percentage change.

© 2025 Cao C et al. *JAMA Network Open*.

**eTable 3. Incidence of oropharyngeal cancer in the US by stage (overall and age stratified), 2006-2021.**

| | Incidence | N | SEER Population |
|---|---|---|---|
| Localized (2006-2021) | | | |
| $APC_{localized}$ (2006-2021)=-0.2%(-1.0% to 0.6%) P = .64 | | | |
| $AAPC_{localized}$ =-0.2%; 95% CI, -1.0% to 0.6%; P = .64 | | | |
| 2006 | 0.6 | 801 | 123,064,363 |
| 2007 | 0.7 | 890 | 124,343,542 |
| 2008 | 0.7 | 893 | 125,772,118 |
| 2009 | 0.7 | 893 | 127,188,118 |
| 2010 | 0.7 | 889 | 128,530,337 |
| 2011 | 0.7 | 968 | 129,847,860 |
| 2012 | 0.7 | 949 | 131,125,146 |
| 2013 | 0.7 | 997 | 132,308,152 |
| 2014 | 0.7 | 1051 | 133,582,353 |
| 2015 | 0.6 | 972 | 134,860,706 |
| 2016 | 0.7 | 1040 | 136,028,149 |
| 2017 | 0.6 | 998 | 137,008,306 |
| 2018 | 0.7 | 1095 | 137,794,939 |
| 2019 | 0.7 | 1066 | 138,472,337 |
| 2020 | 0.6 | 997 | 138,936,824 |
| 2021 | 0.7 | 1149 | 138,818,265 |
| | | | |

© 2025 Cao C et al. *JAMA Network Open*.

| | Incidence | N | SEER Population |
|---|---|---|---|
| Regional (2006-2021) | | | |
| $APC_{regional}$ (2006-2019)=2.4%(2.0% to 2.8%) P < .001 | | | |
| $APC_{regional}$ (2019-2021)=-1.5%(-8.8% to 6.4%) P = .67 | | | |
| $AAPC_{regional}$ =1.9%; 95% CI, 0.9% to 2.9%; P < 0.001 | | | |
| 2006 | 2.3 | 2,856 | 123,064,363 |
| 2007 | 2.4 | 3,117 | 124,343,542 |
| 2008 | 2.5 | 3,287 | 125,772,118 |
| 2009 | 2.6 | 3,542 | 127,188,118 |
| 2010 | 2.5 | 3,520 | 128,530,337 |
| 2011 | 2.6 | 3,709 | 129,847,860 |
| 2012 | 2.6 | 3,776 | 131,125,146 |
| 2013 | 2.7 | 3,991 | 132,308,152 |
| 2014 | 2.8 | 4,253 | 133,582,353 |
| 2015 | 2.9 | 4,480 | 134,860,706 |
| 2016 | 3.1 | 4,845 | 136,028,149 |
| 2017 | 3.0 | 4,904 | 137,008,306 |
| 2018 | 3.1 | 5,112 | 137,794,939 |
| 2019 | 3.2 | 5,401 | 138,472,337 |
| 2020 | 3.1 | 5,169 | 138,936,824 |
| 2021 | 3.1 | 5,286 | 138,818,265 |
| | | | |

© 2025 Cao C et al. *JAMA Network Open*.

| | Incidence | N | SEER Population |
|---|---|---|---|
| Distant (2006-2021) | | | |
| $APC_{distant}$ (2006-2014)=1.7%(-1.7% to 5.1%) P = .30 | | | |
| $APC_{distant}$ (2014-2021)=-9.2%(-12.8% to -5.4%) P < 0.001 | | | |
| $AAPC_{distant}$ =-3.5%; 95% CI, -5.8% to -1.3%; P = .002 | | | |
| 2006 | 0.7 | 836 | 123,064,363 |
| 2007 | 0.7 | 943 | 124,343,542 |
| 2008 | 0.8 | 1,042 | 125,772,118 |
| 2009 | 0.8 | 1,100 | 127,188,118 |
| 2010 | 0.7 | 975 | 128,530,337 |
| 2011 | 0.7 | 1,048 | 129,847,860 |
| 2012 | 0.8 | 1,159 | 131,125,146 |
| 2013 | 0.8 | 1,256 | 132,308,152 |
| 2014 | 0.9 | 1,288 | 133,582,353 |
| 2015 | 0.8 | 1,224 | 134,860,706 |
| 2016 | 0.6 | 926 | 136,028,149 |
| 2017 | 0.6 | 893 | 137,008,306 |
| 2018 | 0.5 | 756 | 137,794,939 |
| 2019 | 0.5 | 787 | 138,472,337 |
| 2020 | 0.4 | 748 | 138,936,824 |
| 2021 | 0.5 | 813 | 138,818,265 |
| | | | |

© 2025 Cao C et al. *JAMA Network Open*.

| | Incidence | N | SEER Population |
|---|---|---|---|
| Age <65 | | | |
| Localized (2006-2021) | | | |
| $APC_{age <65, localized}$ (2006-2021)=-1.0%(-1.9% to 0.0%) P = .05 | | | |
| $AAPC_{age <65, localized}$ =-1.0%; 95% CI, -1.9% to 0.0%; P = .05 | | | |
| 2006 | 0.4 | 504 | 109,064,060 |
| 2007 | 0.5 | 556 | 110,084,040 |
| 2008 | 0.4 | 523 | 111,140,930 |
| 2009 | 0.4 | 513 | 112,205,761 |
| 2010 | 0.4 | 495 | 113,197,288 |
| 2011 | 0.4 | 563 | 114,159,908 |
| 2012 | 0.4 | 518 | 114,763,599 |
| 2013 | 0.4 | 541 | 115,375,732 |
| 2014 | 0.4 | 544 | 116,068,236 |
| 2015 | 0.4 | 511 | 116,765,266 |
| 2016 | 0.4 | 536 | 117,360,675 |
| 2017 | 0.4 | 523 | 117,779,934 |
| 2018 | 0.4 | 529 | 117,982,336 |
| 2019 | 0.4 | 515 | 118,057,077 |
| 2020 | 0.3 | 493 | 117,938,549 |
| 2021 | 0.4 | 537 | 117,273,851 |
| | | | |

© 2025 Cao C et al. *JAMA Network Open*.

| | Incidence | N | SEER Population |
|---|---|---|---|
| Regional (2006-2021) | | | |
| $APC_{age <65, regional}$ (2006-2021)=1.0%(0.5% to 1.5%) P = .001 | | | |
| $AAPC_{age <65, regional}$ =1.0%; 95% CI, 0.5% to 1.5%; P = .001 | | | |
| 2006 | 1.7 | 2,039 | 109,064,060 |
| 2007 | 1.8 | 2,214 | 110,084,040 |
| 2008 | 1.9 | 2,306 | 111,140,930 |
| 2009 | 2.0 | 2,518 | 112,205,761 |
| 2010 | 1.9 | 2,460 | 113,197,288 |
| 2011 | 1.9 | 2,591 | 114,159,908 |
| 2012 | 1.9 | 2,515 | 114,763,599 |
| 2013 | 1.9 | 2,634 | 115,375,732 |
| 2014 | 1.9 | 2,709 | 116,068,236 |
| 2015 | 2.0 | 2,836 | 116,765,266 |
| 2016 | 2.1 | 2,955 | 117,360,675 |
| 2017 | 2.1 | 2,992 | 117,779,934 |
| 2018 | 2.0 | 2,965 | 117,982,336 |
| 2019 | 2.2 | 3,183 | 118,057,077 |
| 2020 | 2.0 | 2,903 | 117,938,549 |
| 2021 | 2.0 | 2,917 | 117,273,851 |
| | | | |

© 2025 Cao C et al. *JAMA Network Open*.

| | Incidence | N | SEER Population |
|---|---|---|---|
| Distant (2006-2021) | | | |
| $APC_{age <65, distant}$ (2006-2014)=2.3%(-0.8% to 5.6%) P = .13 | | | |
| $APC_{age <65, distant}$ (2014-2019)=-18.8%(-25.8% to -11.1%) P = .001 | | | |
| $APC_{age <65, distant}$ (2019-2021)=15.1%(-13.5% to 53.2%) P = .29 | | | |
| $AAPC_{age <65, distant}$ =-3.8%; 95% CI, -7.9% to 0.5%; P = .09 | | | |
| 2006 | 0.5 | 549 | 109,064,060 |
| 2007 | 0.5 | 607 | 110,084,040 |
| 2008 | 0.5 | 678 | 111,140,930 |
| 2009 | 0.6 | 725 | 112,205,761 |
| 2010 | 0.5 | 631 | 113,197,288 |
| 2011 | 0.5 | 676 | 114,159,908 |
| 2012 | 0.5 | 736 | 114,763,599 |
| 2013 | 0.6 | 799 | 115,375,732 |
| 2014 | 0.6 | 802 | 116,068,236 |
| 2015 | 0.5 | 746 | 116,765,266 |
| 2016 | 0.4 | 515 | 117,360,675 |
| 2017 | 0.3 | 490 | 117,779,934 |
| 2018 | 0.3 | 412 | 117,982,336 |
| 2019 | 0.2 | 366 | 118,057,077 |
| 2020 | 0.2 | 370 | 117,938,549 |
| 2021 | 0.3 | 410 | 117,273,851 |
| | | | |

© 2025 Cao C et al. *JAMA Network Open*.

| | Incidence | N | SEER Population |
|---|---|---|---|
| Age ≥65 | | | |
| Localized | | | |
| $APC_{age\ \geq 65,\ localized}$ (2006-2008)=9.9%(-8.7% to 32.3%) P = .29 | | | |
| $APC_{age\ \geq 65,\ localized}$ (2008-2021)=0.4%(-0.5% to 1.4%) P = .36 | | | |
| $AAPC_{age\ \geq 65,\ localized}$ =1.6%; 95% CI, -0.7% to 4.0%; P = .17 | | | |
| 2006 | 2.1 | 297 | 14,000,303 |
| 2007 | 2.4 | 334 | 14,259,502 |
| 2008 | 2.5 | 370 | 14,631,188 |
| 2009 | 2.5 | 380 | 14,982,357 |
| 2010 | 2.6 | 394 | 15,333,049 |
| 2011 | 2.6 | 405 | 15,687,952 |
| 2012 | 2.7 | 431 | 16,361,547 |
| 2013 | 2.7 | 456 | 16,932,420 |
| 2014 | 2.8 | 507 | 17,514,117 |
| 2015 | 2.5 | 461 | 18,095,440 |
| 2016 | 2.7 | 504 | 18,667,474 |
| 2017 | 2.5 | 475 | 19,228,372 |
| 2018 | 2.9 | 566 | 19,812,603 |
| 2019 | 2.7 | 551 | 20,415,260 |
| 2020 | 2.4 | 504 | 20,998,275 |
| 2021 | 2.9 | 612 | 21,544,414 |
| | | | |

© 2025 Cao C et al. *JAMA Network Open*.

| | Incidence | N | SEER Population |
|---|---|---|---|
| Regional (2006-2021) | | | |
| $APC_{age \geqslant 65, regional}$ (2006-2018)=4.9%(4.3% to 5.5%) P < .001 | | | |
| $APC_{age \geqslant 65, regional}$ (2018-2021)=0.8%(-4.0% to 5.8%) P = .74 | | | |
| $AAPC_{age \geqslant 65, regional}$ =4.1%; 95% CI, 3.1% to 5.1%; P < .001 | | | |
| 2006 | 5.8 | 817 | 14,000,303 |
| 2007 | 6.3 | 903 | 14,259,502 |
| 2008 | 6.7 | 981 | 14,631,188 |
| 2009 | 6.8 | 1,024 | 14,982,357 |
| 2010 | 6.9 | 1,060 | 15,333,049 |
| 2011 | 7.0 | 1,118 | 15,687,952 |
| 2012 | 7.6 | 1,261 | 16,361,547 |
| 2013 | 7.9 | 1,357 | 16,932,420 |
| 2014 | 8.6 | 1,544 | 17,514,117 |
| 2015 | 8.9 | 1,644 | 18,095,440 |
| 2016 | 9.9 | 1,890 | 18,667,474 |
| 2017 | 9.7 | 1,912 | 19,228,372 |
| 2018 | 10.6 | 2,147 | 19,812,603 |
| 2019 | 10.5 | 2,218 | 20,415,260 |
| 2020 | 10.5 | 2,266 | 20,998,275 |
| 2021 | 10.6 | 2,369 | 21,544,414 |
| | | | |

© 2025 Cao C et al. *JAMA Network Open*.

| | Incidence | N | SEER Population |
|---|---|---|---|
| Distant (2006-2021) | | | |
| $APC_{age\ \geqslant 65,\ distant}$ (2006-2014)=2.0%(-0.7% to 4.7%) P = .14 | | | |
| $APC_{age\ \geqslant 65,\ distant}$ (2014-2021)=-6.3%(-9.3% to -3.1%) P = .001 | | | |
| $AAPC_{age\ \geqslant 65,\ distant}$ =-2.0%; 95% CI, -3.8% to -0.1%; P = .04 | | | |
| 2006 | 2.1 | 287 | 14,000,303 |
| 2007 | 2.4 | 336 | 14,259,502 |
| 2008 | 2.5 | 364 | 14,631,188 |
| 2009 | 2.5 | 375 | 14,982,357 |
| 2010 | 2.2 | 344 | 15,333,049 |
| 2011 | 2.4 | 372 | 15,687,952 |
| 2012 | 2.6 | 423 | 16,361,547 |
| 2013 | 2.6 | 457 | 16,932,420 |
| 2014 | 2.8 | 486 | 17,514,117 |
| 2015 | 2.6 | 478 | 18,095,440 |
| 2016 | 2.2 | 411 | 18,667,474 |
| 2017 | 2.1 | 403 | 19,228,372 |
| 2018 | 1.7 | 344 | 19,812,603 |
| 2019 | 2.0 | 421 | 20,415,260 |
| 2020 | 1.8 | 378 | 20,998,275 |
| 2021 | 1.8 | 403 | 21,544,414 |

Incidence per 100,000 persons, age-adjusted to the 2000 US standard population (19 age groups - Census P25-1130). US, United States; y, years; APC, annual percentage change; AAPC, average annual percentage change.

© 2025 Cao C et al. *JAMA Network Open*.

**eTable 4. 10-Year prevalence of oropharyngeal cancer in the US.**

| | Overall | | Age <65y | | Age ≥65y | | Male | | Female | |
|---|---|---|---|---|---|---|---|---|---|---|
| | 10-year duration Prevalence | 10-year Count | 10-year duration Prevalence | 10-year Count | 10-year duration Prevalence | 10-year Count | 10-year duration Prevalence | 10-year Count | 10-year duration Prevalence | 10-year Count |
| 2012 | 0.02422% | 31,607.4 | 0.01816% | 23,691.9 | 0.00608% | 7,929.7 | 0.03879% | 24,987.4 | 0.01002% | 6,620.0 |
| 2013 | 0.02530% | 33,326.3 | 0.01881% | 24,777.8 | 0.00650% | 8,565.6 | 0.04066% | 26,464.1 | 0.01030% | 6,863.0 |
| 2014 | 0.02639% | 35,084.4 | 0.01946% | 25,872.5 | 0.00694% | 9,228.6 | 0.04251% | 27,959.1 | 0.01061% | 7,126.4 |
| 2015 | 0.02760% | 37,041.8 | 0.02006% | 26,925.8 | 0.00755% | 10,134.4 | 0.04461% | 29,651.6 | 0.01091% | 7,391.0 |
| 2016 | 0.02868% | 38,842.6 | 0.02059% | 27,887.6 | 0.00810% | 10,975.1 | 0.04665% | 31,318.6 | 0.01102% | 7,526.0 |
| 2017 | 0.02981% | 40,702.1 | 0.02100% | 28,670.5 | 0.00882% | 12,047.6 | 0.04870% | 32,985.5 | 0.01122% | 7,717.6 |
| 2018 | 0.03067% | 42,142.9 | 0.02131% | 29,282.5 | 0.01048% | 14,399.4 | 0.05018% | 34,235.4 | 0.01143% | 7,908.1 |
| 2019 | 0.03167% | 43,745.3 | 0.02155% | 29,770.4 | 0.01013% | 13,994.6 | 0.05185% | 35,598.2 | 0.01173% | 8,147.4 |
| 2020 | 0.03257% | 45,176.1 | 0.02175% | 30,175.0 | 0.01083% | 15,023.8 | 0.05343% | 36,859.7 | 0.01193% | 8,316.4 |
| 2021 | 0.03323% | 46,146.7 | 0.02181% | 30,294.2 | 0.01144% | 15,881.3 | 0.05470% | 37,784.6 | 0.01198% | 8,362.0 |

*all calculation are based on SEER 22 (exl IL and MA). Prevalence date is Jan 1st of each year. US, United States; y, years.

© 2025 Cao C et al. *JAMA Network Open*.

**eTable 5. Initial treatment for oropharyngeal cancer in the US.**

| | | | Any Treatment (%) | | | Single Treatment (%) | | | | Multiple Treatments (%) | |
|---|---|---|---|---|---|---|---|---|---|---|---|
| | N | No Treatment *(%) | Surgery | Chemotherapy | Radiation | Only 1 Treatment | Surgery Only | Chemotherapy Only | Radiation Only | More than 1 Treatment | Chemoradiotherapy |
| Overall | | | | | | | | | | | |
| 2006 | 4,754 | 1,071(22.5) | 1,590 (33.4) | 2,929 (61.6) | 2,924 (61.5) | 962 (20.2) | 45 (0.9) | 573 (12.1) | 344 (7.2) | 2,721 (57.2) | 2,215 (46.6) |
| 2007 | 5,167 | 1,107 (21.4) | 1,738 (33.6) | 3,341 (64.7) | 3,182 (61.6) | 1,033 (20.0) | 55 (1.1) | 664 (12.9) | 314 (6.1) | 3,027 (58.6) | 2,518 (48.7) |
| 2008 | 5,446 | 1,112 (20.4) | 1,752 (32.2) | 3,578 (65.7) | 3,346 (61.4) | 1,178 (21.6) | 44 (0.8) | 795 (14.6) | 339 (6.2) | 3,156 (58.0) | 2,634 (48.4) |
| 2009 | 5,837 | 1,233 (21.1) | 1,844 (31.6) | 3,872 (66.3) | 3,600 (61.7) | 1,196 (20.5) | 51 (0.9) | 831 (14.2) | 314 (5.4) | 3,408 (58.4) | 2,919 (50.0) |
| 2010 | 5,688 | 1,154 (20.3) | 1,994 (35.1) | 3,788 (66.6) | 3,598 (63.3) | 1,077 (18.9) | 53 (0.9) | 727 (12.8) | 297 (5.2) | 3,457 (60.8) | 2,905 (51.1) |
| 2011 | 6,017 | 1,283 (21.3) | 2,216 (36.8) | 3,915 (65.1) | 3,736 (62.1) | 1,129 (18.8) | 51 (0.8) | 763 (12.7) | 315 (5.2) | 3,605 (59.9) | 2,968 (49.3) |
| 2012 | 6,159 | 1,282 (20.8) | 2,214 (35.9) | 4,032 (65.5) | 3,841 (62.4) | 1,176 (19.1) | 51 (0.8) | 812 (13.2) | 313 (5.2) | 3,701 (60.1) | 3,047 (49.5) |
| 2013 | 6,504 | 1,384 (21.3) | 2,310 (35.5) | 4,153 (63.9) | 4,075 (62.7) | 1,257 (19.3) | 48 (0.7) | 806 (12.4) | 403 (6.2) | 3,863 (59.4) | 3,156 (48.5) |
| 2014 | 6,877 | 1,437 (20.9) | 2,387 (34.7) | 4,314 (62.7) | 4,343 (63.2) | 1,389 (20.2) | 56 (0.8) | 862 (12.5) | 471 (6.8) | 4,051 (58.9) | 3,273 (47.6) |
| 2015 | 6,947 | 1,496 (21.5) | 2,457 (35.4) | 4,337 (62.4) | 4,379 (63.0) | 1,313 (18.9) | 70 (1.0) | 814 (11.7) | 429 (6.2) | 4,138 (59.6) | 3,335 (48.0) |
| 2016 | 7,148 | 1,544 (21.6) | 2,435 (34.1) | 4,342 (60.7) | 4,540 (63.5) | 1,371 (19.2) | 74 (1.0) | 788 (11.0) | 509 (7.1) | 4,233 (59.2) | 3,352 (46.9) |
| 2017 | 7,108 | 1,598 (22.5) | 2,337 (32.9) | 4,300 (60.5) | 4,408 (62.0) | 1,390 (19.6) | 82 (1.2) | 812 (11.4) | 496 (7.0) | 4,120 (58.0) | 3,280 (46.1) |
| 2018 | 7,275 | 1,586 (21.8) | 2,417 (33.2) | 4,433 (60.9) | 4,549 (62.5) | 1,482 (20.4) | 88 (1.2) | 840 (11.5) | 554 (7.6) | 4,207 (57.8) | 3,381 (46.5) |
| 2019 | 7,523 | 1,611 (21.4) | 2,635 (35.0) | 4,814 (64.0) | 4,692 (62.4) | 1,382 (18.4) | 83 (1.1) | 872 (11.6) | 427 (5.7) | 4,530 (60.2) | 3,677 (48.9) |
| 2020 | 7,162 | 1,428 (19.9) | 2,400 (33.5) | 4,671 (65.2) | 4,386 (61.2) | 1,455 (20.3) | 105 (1.5) | 943 (13.2) | 407 (5.7) | 4,279 (59.7) | 3,428 (47.9) |
| 2021 | 7,495 | 1,621 (21.6) | 1,480 (19.7) | 4,803 (64.1) | 4,570 (61.0) | 1,647 (22.0) | 85 (1.1) | 1051 (14.0) | 511 (6.8) | 4,227 (56.4) | 3,584 (47.8) |
| | | | | | | | | | | | |

© 2025 Cao C et al. *JAMA Network Open*.

| | | | | Any Treatment (%) | | | Single Treatment (%) | | | Multiple Treatments (%) | |
|---|---|---|---|---|---|---|---|---|---|---|---|
| | N | No Treatment *(%) | Surgery | Chemotherapy | Radiation | Only 1 Treatment | Surgery Only | Chemotherapy Only | Radiation Only | More than 1 Treatment | Chemoradiotherapy |
| Age<65y | | | | | | | | | | | |
| 2006 | 3,241 | 623 (19.2) | 1,255 (38.7) | 2,136 (65.9) | 2,086 (64.4) | 599 (18.5) | 40 (1.2) | 380 (11.7) | 179 (5.5) | 2,019 (62.3) | 1,644 (50.7) |
| 2007 | 3,506 | 648 (18.5) | 1,317 (37.6) | 2,401 (68.5) | 2,257 (64.4) | 645 (18.4) | 44 (1.3) | 432 (12.3) | 169 (4.8) | 2,213 (63.1) | 1,844 (52.6) |
| 2008 | 3,634 | 647 (17.8) | 1,349 (37.1) | 2,541 (69.9) | 2,314 (63.7) | 707 (19.5) | 28 (0.8) | 529 (14.6) | 150 (4.1) | 2,280 (62.7) | 1,896 (52.2) |
| 2009 | 3,937 | 687 (17.4) | 1,416 (36.0) | 2,808 (71.3) | 2,565 (65.2) | 746 (18.9) | 40 (1.0) | 547 (13.9) | 159 (4.0) | 2,504 (63.6) | 2,163 (54.9) |
| 2010 | 3,754 | 641 (17.1) | 1,507 (40.1) | 2,648 (70.5) | 2,488 (66.3) | 644 (17.2) | 40 (1.1) | 458 (12.2) | 146 (3.9) | 2,469 (65.8) | 2,063 (55.0) |
| 2011 | 3,992 | 724 (18.1) | 1,670 (41.8) | 2,767 (69.3) | 2,589 (64.9) | 687 (17.2) | 36 (0.9) | 492 (12.3) | 159 (4.0) | 2,581 (64.7) | 2,124 (53.2) |
| 2012 | 3,948 | 708 (17.9) | 1,595 (40.4) | 2,751 (69.7) | 2,554 (64.7) | 697 (17.7) | 30 (0.8) | 521 (13.2) | 146 (3.7) | 2,543 (64.4) | 2,095 (53.1) |
| 2013 | 4,117 | 723 (17.6) | 1,678 (40.8) | 2,845 (69.1) | 2,700 (65.6) | 729 (17.7) | 36 (0.9) | 514 (12.5) | 179 (4.3) | 2,665 (64.7) | 2,187 (53.1) |
| 2014 | 4,211 | 753 (17.9) | 1,639 (38.9) | 2,871 (68.2) | 2,762 (65.6) | 759 (18.0) | 32 (0.8) | 526 (12.5) | 201 (4.8) | 2,699 (64.1) | 2,207 (52.4) |
| 2015 | 4,249 | 811 (19.1) | 1,687 (39.7) | 2,845 (67.0) | 2,778 (65.4) | 695 (16.4) | 42 (1.0) | 481 (11.3) | 172 (4.0) | 2,743 (64.6) | 2,227 (52.4) |
| 2016 | 4,181 | 788 (18.8) | 1,608 (38.5) | 2,725 (65.2) | 2,712 (64.9) | 740 (17.7) | 59 (1.4) | 482 (11.5) | 199 (4.8) | 2,653 (63.5) | 2,103 (50.3) |
| 2017 | 4,180 | 840 (20.1) | 1,552 (37.1) | 2,728 (65.3) | 2,672 (63.9) | 702 (16.8) | 45 (1.1) | 474 (11.3) | 183 (4.4) | 2,638 (63.1) | 2,105 (50.4) |
| 2018 | 4,060 | 771 (19.0) | 1,534 (37.8) | 2,688 (66.2) | 2,603 (64.1) | 742 (18.3) | 63 (1.6) | 490 (12.1) | 189 (4.7) | 2,547 (62.7) | 2,065 (50.9) |
| 2019 | 4,198 | 789 (18.8) | 1,648 (39.3) | 2,886 (68.7) | 2,726 (64.9) | 652 (15.5) | 46 (1.1) | 466 (11.1) | 140 (3.3) | 2,757 (65.7) | 2,249 (53.6) |
| 2020 | 3,878 | 663 (17.1) | 1,436 (37.0) | 2,699 (69.6) | 2,486 (64.1) | 705 (18.2) | 51 (1.3) | 506 (13.0) | 148 (3.8) | 2,510 (64.7) | 2,021 (52.1) |
| 2021 | 3,970 | 756 (19.0) | 910 (22.9) | 2,704 (68.1) | 2,489 (62.7) | 817 (20.6) | 48 (1.2) | 572 (14.4) | 197 (5.0) | 2,397 (60.4) | 2,027 (51.1) |
| | | | | | | | | | | | |

© 2025 Cao C et al. *JAMA Network Open*.

| | | | | Any Treatment (%) | | | Single Treatment (%) | | | Multiple Treatments (%) | |
|---|---|---|---|---|---|---|---|---|---|---|---|
| | N | No Treatment *(%) | Surgery | Chemotherapy | Radiation | Only 1 Treatment | Surgery Only | Chemotherapy Only | Radiation Only | More than 1 Treatment | Chemoradiotherapy |
| Age≥65y | | | | | | | | | | | |
| 2006 | 1,513 | 448 (29.6) | 335 (22.1) | 793 (52.4) | 838 (55.4) | 363 (24.0) | 5 (0.3) | 193 (12.8) | 165 (10.9) | 702 (46.4) | 571 (37.7) |
| 2007 | 1,661 | 459 (27.6) | 421 (25.3) | 940 (56.6) | 925 (55.7) | 388 (23.4) | 11 (0.7) | 232 (14.0) | 145 (8.7) | 814 (49.0) | 674 (40.6) |
| 2008 | 1,812 | 465 (25.7) | 403 (22.2) | 1,037 (57.2) | 1,032 (57.0) | 471 (26.0) | 16 (0.9) | 266 (14.7) | 189 (10.4) | 876 (48.3) | 738 (40.7) |
| 2009 | 1,900 | 546 (28.7) | 428 (22.5) | 1,064 (56.0) | 1,035 (54.5) | 450 (23.7) | 11 (0.6) | 284 (14.9) | 155 (8.2) | 904 (47.6) | 756 (39.8) |
| 2010 | 1,934 | 513 (26.5) | 487 (25.2) | 1,140 (58.9) | 1,110 (57.4) | 433 (22.4) | 13 (0.7) | 269 (13.9) | 151 (7.8) | 988 (51.1) | 842 (43.5) |
| 2011 | 2,025 | 559 (27.6) | 546 (27.0) | 1,148 (56.7) | 1,147 (56.6) | 442 (21.8) | 15 (0.7) | 271 (13.4) | 156 (7.7) | 1,024 (50.6) | 844 (41.7) |
| 2012 | 2,211 | 574 (26.0) | 619 (28.0) | 1,281 (57.9) | 1,287 (58.2) | 479 (21.7) | 21 (0.9) | 291 (13.2) | 167 (7.6) | 1,158 (52.4) | 952 (43.1) |
| 2013 | 2,387 | 661 (27.7) | 632 (26.5) | 1,308 (54.8) | 1,375 (57.6) | 528 (22.1) | 12 (0.5) | 292 (12.2) | 224 (9.4) | 1,198 (50.2) | 969 (40.6) |
| 2014 | 2,666 | 684 (25.7) | 748 (28.1) | 1,443 (54.1) | 1,581 (59.3) | 630 (23.6) | 24 (0.9) | 336 (12.6) | 270 (10.1) | 1,352 (50.7) | 1,066 (40.0) |
| 2015 | 2,698 | 685 (25.4) | 770 (28.5) | 1,492 (55.3) | 1,601 (59.3) | 618 (22.9) | 28 (1.0) | 333 (12.3) | 257 (9.5) | 1,395 (51.7) | 1,108 (41.1) |
| 2016 | 2,967 | 756 (25.5) | 827 (27.9) | 1,617 (54.5) | 1,828 (61.6) | 631 (21.3) | 15 (0.5) | 306 (10.3) | 310 (10.4) | 1,580 (53.3) | 1,249 (42.1) |
| 2017 | 2,928 | 758 (25.9) | 785 (26.8) | 1,572 (53.7) | 1,736 (59.3) | 688 (23.5) | 37 (1.3) | 338 (11.5) | 313 (10.7) | 1,482 (50.6) | 1,175 (40.1) |
| 2018 | 3,215 | 815 (25.3) | 883 (27.5) | 1,745 (54.3) | 1,946 (60.5) | 740 (23.0) | 25 (0.8) | 350 (10.9) | 365 (11.4) | 1,660 (51.6) | 1,316 (40.9) |
| 2019 | 3,325 | 822 (24.7) | 987 (29.7) | 1,928 (58.0) | 1,966 (59.1) | 730 (22.0) | 37 (1.1) | 406 (12.2) | 287 (8.6) | 1,773 (53.3) | 1,428 (42.9) |
| 2020 | 3,284 | 765 (23.3) | 964 (29.4) | 1,972 (60.0) | 1,900 (57.9) | 750 (22.8) | 54 (1.6) | 437 (13.3) | 259 (7.9) | 1,769 (53.9) | 1,407 (42.8) |
| 2021 | 3,525 | 865 (24.5) | 570 (16.2) | 2,099 (59.5) | 2,081 (59.0) | 830 (23.5) | 37 (1.0) | 479 (13.6) | 314 (8.9) | 1,830 (51.9) | 1,557 (44.2) |
| | | | | | | | | | | | |

© 2025 Cao C et al. *JAMA Network Open*.

| | | | | Any Treatment (%) | | | Single Treatment (%) | | | Multiple Treatments (%) | |
|---|---|---|---|---|---|---|---|---|---|---|---|
| | N | No Treatment *(%) | Surgery | Chemotherapy | Radiation | Only 1 Treatment | Surgery Only | Chemotherapy Only | Radiation Only | More than 1 Treatment | Chemoradiotherapy |
| Male | | | | | | | | | | | |
| 2006 | 3,694 | 749 (20.3) | 1,288 (34.9) | 2,383 (64.5) | 2,358 (63.8) | 723 (19.6) | 37 (1.0) | 434 (11.7) | 252 (6.8) | 2,222 (60.2) | 1,833 (49.6) |
| 2007 | 4,049 | 787 (19.4) | 1,411 (34.8) | 2,711 (67.0) | 2,603 (64.3) | 775 (19.1) | 48 (1.2) | 491 (12.1) | 236 (5.8) | 2,487 (61.4) | 2,100 (51.9) |
| 2008 | 4,289 | 797 (18.6) | 1,442 (33.6) | 2,937 (68.5) | 2,727 (63.6) | 889 (20.7) | 36 (0.8) | 609 (14.2) | 244 (5.7) | 2,603 (60.7) | 2,208 (51.5) |
| 2009 | 4,639 | 914 (19.7) | 1,506 (32.5) | 3,171 (68.4) | 2,955 (63.7) | 905 (19.5) | 41 (0.9) | 629 (13.6) | 235 (5.1) | 2,820 (60.8) | 2,442 (52.6) |
| 2010 | 4,440 | 817 (18.4) | 1,634 (36.8) | 3,072 (69.2) | 2,896 (65.2) | 807 (18.2) | 43 (1.0) | 556 (12.5) | 208 (4.7) | 2,816 (63.4) | 2,388 (53.8) |
| 2011 | 4,759 | 935 (19.6) | 1,805 (37.9) | 3,224 (67.7) | 3,038 (63.8) | 856 (18.0) | 38 (0.8) | 598 (12.6) | 220 (4.6) | 2,968 (62.4) | 2,476 (52.0) |
| 2012 | 4,892 | 941 (19.2) | 1,820 (37.2) | 3,301 (67.5) | 3,122 (63.8) | 920 (18.8) | 39 (0.8) | 644 (13.2) | 237 (4.8) | 3,031 (62.0) | 2,511 (51.3) |
| 2013 | 5,236 | 1,025 (19.6) | 1,914 (36.6) | 3,464 (66.2) | 3,371 (64.4) | 985 (18.8) | 41 (0.8) | 640 (12.2) | 304 (5.8) | 3,226 (61.6) | 2,665 (50.9) |
| 2014 | 5,537 | 1,058 (19.1) | 1,996 (36.0) | 3,592 (64.9) | 3,599 (65.0) | 1,082 (19.5) | 46 (0.8) | 677 (12.2) | 359 (6.5) | 3,397 (61.4) | 2,758 (49.8) |
| 2015 | 5,624 | 1,129 (20.1) | 2,072 (36.8) | 3,603 (64.1) | 3,648 (64.9) | 1,011 (18.0) | 57 (1.0) | 629 (11.2) | 325 (5.8) | 3,484 (61.9) | 2,813 (50.0) |
| 2016 | 5,827 | 1,152 (19.8) | 2,051 (35.2) | 3,657 (62.8) | 3,808 (65.4) | 1,113 (19.1) | 65 (1.1) | 637 (10.9) | 411 (7.1) | 3,562 (61.1) | 2,855 (49.0) |
| 2017 | 5,782 | 1,203 (20.8) | 1,975 (34.2) | 3,604 (62.3) | 3,680 (63.6) | 1,103 (19.1) | 71 (1.2) | 647 (11.2) | 385 (6.7) | 3,476 (60.1) | 2,776 (48.0) |
| 2018 | 5,906 | 1,191 (20.2) | 2,027 (34.3) | 3,704 (62.7) | 3,813 (64.6) | 1,163 (19.7) | 68 (1.2) | 648 (11.0) | 447 (7.6) | 3,552 (60.1) | 2,870 (48.6) |
| 2019 | 6,145 | 1,209 (19.7) | 2,227 (36.2) | 4,049 (65.9) | 3,925 (63.9) | 1,113 (18.1) | 72 (1.2) | 706 (11.5) | 335 (5.5) | 3,823 (62.2) | 3,110 (50.6) |
| 2020 | 5,857 | 1,076 (18.4) | 2,027 (34.6) | 3,934 (67.2) | 3,702 (63.2) | 1,151 (19.7) | 85 (1.5) | 744 (12.7) | 322 (5.5) | 3,630 (62.0) | 2,940 (50.2) |
| 2021 | 6,144 | 1,242 (20.2) | 1,244 (20.2) | 4,052 (66.0) | 3,839 (62.5) | 1,313 (21.4) | 69 (1.1) | 847 (13.8) | 397 (6.5) | 3,589 (58.4) | 3,058 (49.8) |
| | | | | | | | | | | | |

© 2025 Cao C et al. *JAMA Network Open*.

| | | | | Any Treatment (%) | | | Single Treatment (%) | | | Multiple Treatments (%) | |
|---|---|---|---|---|---|---|---|---|---|---|---|
| | N | No Treatment *(%) | Surgery | Chemotherapy | Radiation | Only 1 Treatment | Surgery Only | Chemotherapy Only | Radiation Only | More than 1 Treatment | Chemoradiotherapy |
| Female | | | | | | | | | | | |
| 2006 | 1,060 | 322 (30.4) | 302 (28.5) | 546 (51.5) | 566 (53.4) | 239 (22.5) | 8 (0.8) | 139 (13.1) | 92 (8.7) | 499 (47.1) | 382 (36.0) |
| 2007 | 1,118 | 320 (28.6) | 327 (29.2) | 630 (56.4) | 579 (51.8) | 258 (23.1) | 7 (0.6) | 173 (15.5) | 78 (7.0) | 540 (48.3) | 418 (37.4) |
| 2008 | 1,157 | 315 (27.2) | 310 (26.8) | 641 (55.4) | 619 (53.5) | 289 (25.0) | 8 (0.7) | 186 (16.1) | 95 (8.2) | 553 (47.8) | 426 (36.8) |
| 2009 | 1,198 | 319 (26.6) | 338 (28.2) | 701 (58.5) | 645 (53.8) | 291 (24.3) | 10 (0.8) | 202 (16.9) | 79 (6.6) | 588 (49.1) | 477 (39.8) |
| 2010 | 1,248 | 337 (27.0) | 360 (28.8) | 716 (57.4) | 702 (56.3) | 270 (21.6) | 10 (0.8) | 171 (13.7) | 89 (7.1) | 641 (51.4) | 517 (41.4) |
| 2011 | 1,258 | 348 (27.7) | 411 (32.7) | 691 (54.9) | 698 (55.5) | 273 (21.7) | 13 (1.0) | 165 (13.1) | 95 (7.6) | 637 (50.6) | 492 (39.1) |
| 2012 | 1,267 | 341 (26.9) | 394 (31.1) | 731 (57.7) | 719 (56.7) | 256 (20.2) | 12 (0.9) | 168 (13.3) | 76 (6.0) | 670 (52.9) | 536 (42.3) |
| 2013 | 1,268 | 359 (28.3) | 396 (31.2) | 689 (54.3) | 704 (55.5) | 272 (21.5) | 7 (0.6) | 166 (13.1) | 99 (7.8) | 637 (50.2) | 491 (38.7) |
| 2014 | 1,340 | 379 (28.3) | 391 (29.2) | 722 (53.9) | 744 (55.5) | 307 (22.9) | 10 (0.7) | 185 (13.8) | 112 (8.4) | 654 (48.8) | 515 (38.4) |
| 2015 | 1,323 | 367 (27.7) | 385 (29.1) | 734 (55.5) | 731 (55.3) | 302 (22.8) | 13 (1.0) | 185 (14.0) | 104 (7.9) | 654 (49.4) | 522 (39.5) |
| 2016 | 1,321 | 392 (29.7) | 384 (29.1) | 685 (51.9) | 732 (55.4) | 258 (19.5) | 9 (0.7) | 151 (11.4) | 98 (7.4) | 671 (50.8) | 497 (37.6) |
| 2017 | 1,326 | 395 (29.8) | 362 (27.3) | 696 (52.5) | 728 (54.9) | 287 (21.6) | 11 (0.8) | 165 (12.4) | 111 (8.4) | 644 (48.6) | 504 (38.0) |
| 2018 | 1,369 | 395 (28.9) | 390 (28.5) | 729 (53.3) | 736 (53.8) | 319 (23.3) | 20 (1.5) | 192 (14.0) | 107 (7.8) | 655 (47.8) | 511 (37.3) |
| 2019 | 1,378 | 402 (29.2) | 408 (29.6) | 765 (55.5) | 767 (55.7) | 269 (19.5) | 11 (0.8) | 166 (12.0) | 92 (6.7) | 707 (51.3) | 567 (41.1) |
| 2020 | 1,305 | 352 (27.0) | 373 (28.6) | 737 (56.5) | 684 (52.4) | 304 (23.3) | 20 (1.5) | 199 (15.2) | 85 (6.5) | 649 (49.7) | 488 (37.4) |
| 2021 | 1,351 | 379 (28.1) | 236 (17.5) | 751 (55.6) | 731 (54.1) | 334 (24.7) | 16 (1.2) | 204 (15.1) | 114 (8.4) | 638 (47.2) | 526 (38.9) |
| | | | | | | | | | | | |

© 2025 Cao C et al. *JAMA Network Open*.

| | | | | Any Treatment (%) | | | Single Treatment (%) | | | Multiple Treatments (%) | |
|---|---|---|---|---|---|---|---|---|---|---|---|
| | N | No Treatment *(%) | Surgery | Chemotherapy | Radiation | Only 1 Treatment | Surgery Only | Chemotherapy Only | Radiation Only | More than 1 Treatment | Chemoradiotherapy |
| Metropolitan | | | | | | | | | | | |
| 2006 | 4,092 | 914 (22.3) | 1,350 (33.0) | 2,539 (62.0) | 2,525 (61.7) | 822 (20.1) | 42 (1.0) | 493 (12.0) | 287 (7.0) | 2,356 (57.6) | 1,928 (47.1) |
| 2007 | 4,409 | 914 (20.7) | 1,474 (33.4) | 2,897 (65.7) | 2,738 (62.1) | 893 (20.3) | 44 (1.0) | 581 (13.2) | 268 (6.1) | 2,602 (59.0) | 2,184 (49.5) |
| 2008 | 4,739 | 947 (20.0) | 1,538 (32.5) | 3,153 (66.5) | 2,948 (62.2) | 1,004 (21.2) | 39 (0.8) | 675 (14.2) | 290 (6.1) | 2,788 (58.8) | 2,348 (49.5) |
| 2009 | 5,096 | 1,087 (21.3) | 1,596 (31.3) | 3,371 (66.1) | 3,149 (61.8) | 1,031 (20.2) | 43 (0.8) | 714 (14.0) | 274 (5.4) | 2,978 (58.4) | 2,554 (50.1) |
| 2010 | 4,959 | 1,010 (20.4) | 1,736 (35.0) | 3,302 (66.6) | 3,146 (63.4) | 927 (18.7) | 46 (0.9) | 624 (12.6) | 257 (5.2) | 3,022 (60.9) | 2,545 (51.3) |
| 2011 | 5,222 | 1,094 (20.9) | 1,934 (37.0) | 3,404 (65.2) | 3,254 (62.3) | 997 (19.1) | 46 (0.9) | 665 (12.7) | 286 (5.5) | 3,131 (60.0) | 2,576 (49.3) |
| 2012 | 5,311 | 1,080 (20.3) | 1,899 (35.8) | 3,504 (66.0) | 3,323 (62.6) | 1,036 (19.5) | 45 (0.8) | 722 (13.6) | 269 (5.1) | 3,195 (60.2) | 2,641 (49.7) |
| 2013 | 5,601 | 1,158 (20.7) | 2,017 (36.0) | 3,598 (64.2) | 3,550 (63.4) | 1,073 (19.2) | 40 (0.7) | 686 (12.2) | 347 (6.2) | 3,370 (60.2) | 2,745 (49.0) |
| 2014 | 5,936 | 1,225 (20.6) | 2,078 (35.0) | 3,732 (62.9) | 3,777 (63.6) | 1,179 (19.9) | 52 (0.9) | 734 (12.4) | 393 (6.6) | 3,532 (59.5) | 2,850 (48.0) |
| 2015 | 6,009 | 1,288 (21.4) | 2,124 (35.3) | 3,732 (62.1) | 3,807 (63.4) | 1,125 (18.7) | 62 (1.0) | 685 (11.4) | 378 (6.3) | 3,596 (59.8) | 2,880 (47.9) |
| 2016 | 6,193 | 1,344 (21.7) | 2,099 (33.9) | 3,760 (60.7) | 3,944 (63.7) | 1,166 (18.8) | 58 (0.9) | 670 (10.8) | 438 (7.1) | 3,683 (59.5) | 2,913 (47.0) |
| 2017 | 6,190 | 1,382 (22.3) | 2,032 (32.8) | 3,757 (60.7) | 3,852 (62.2) | 1,199 (19.4) | 73 (1.2) | 702 (11.3) | 424 (6.8) | 3,609 (58.3) | 2,874 (46.4) |
| 2018 | 6,289 | 1,338 (21.3) | 2,089 (33.2) | 3,853 (61.3) | 3,967 (63.1) | 1,277 (20.3) | 80 (1.3) | 719 (11.4) | 478 (7.6) | 3,674 (58.4) | 2,949 (46.9) |
| 2019 | 6,465 | 1,391 (21.5) | 2,256 (34.9) | 4,134 (63.9) | 4,050 (62.6) | 1,156 (17.9) | 69 (1.1) | 730 (11.3) | 357 (5.5) | 3,918 (60.6) | 3,179 (49.2) |
| 2020 | 6,178 | 1,228 (19.9) | 2,100 (34.0) | 4,018 (65.0) | 3,820 (61.8) | 1,217 (19.7) | 85 (1.4) | 781 (12.6) | 351 (5.7) | 3,733 (60.4) | 2,973 (48.1) |
| 2021 | 6,389 | 1,351 (21.1) | 1,302 (20.4) | 4,105 (64.3) | 3,926 (61.4) | 1,401 (21.9) | 74 (1.2) | 890 (13.9) | 437 (6.8) | 3,637 (56.9) | 3,067 (48.0) |
| | | | | | | | | | | | |

© 2025 Cao C et al. *JAMA Network Open*.

| | | | | Any Treatment (%) | | | Single Treatment (%) | | | Multiple Treatments (%) | |
|---|---|---|---|---|---|---|---|---|---|---|---|
| | N | No Treatment *(%) | Surgery | Chemotherapy | Radiation | Only 1 Treatment | Surgery Only | Chemotherapy Only | Radiation Only | More than 1 Treatment | Chemoradiotherapy |
| Nonmetropolitan | | | | | | | | | | | |
| 2006 | 659 | 155 (23.5) | 240 (36.4) | 389 (59.0) | 398 (60.4) | 140 (21.2) | 3 (0.5) | 80 (12.1) | 57 (8.6) | 364 (55.2) | 286 (43.4) |
| 2007 | 753 | 193 (25.6) | 263 (34.9) | 440 (58.4) | 440 (58.4) | 139 (18.5) | 11 (1.5) | 82 (10.9) | 46 (6.1) | 421 (55.9) | 331 (44.0) |
| 2008 | 707 | 165 (23.3) | 214 (30.3) | 425 (60.1) | 398 (56.3) | 174 (24.6) | 5 (0.7) | 120 (17.0) | 49 (6.9) | 368 (52.1) | 286 (40.5) |
| 2009 | 739 | 145 (19.6) | 247 (33.4) | 501 (67.8) | 450 (60.9) | 165 (22.3) | 8 (1.1) | 117 (15.8) | 40 (5.4) | 429 (58.1) | 365 (49.4) |
| 2010 | 723 | 144 (19.9) | 256 (35.4) | 482 (66.7) | 447 (61.8) | 147 (20.3) | 7 (1.0) | 102 (14.1) | 38 (5.3) | 432 (59.8) | 357 (49.4) |
| 2011 | 790 | 188 (23.8) | 281 (35.6) | 509 (64.4) | 478 (60.5) | 131 (16.6) | 5 (0.6) | 98 (12.4) | 28 (3.5) | 471 (59.6) | 390 (49.4) |
| 2012 | 842 | 201 (23.9) | 314 (37.3) | 524 (62.2) | 514 (61.0) | 138 (16.4) | 6 (0.7) | 89 (10.6) | 43 (5.1) | 503 (59.7) | 403(47.9) |
| 2013 | 897 | 224 (25.0) | 291 (32.4) | 553 (61.6) | 521 (58.1) | 183 (20.4) | 8 (0.9) | 120 (13.4) | 55 (6.1) | 490 (54.6) | 409 (45.6) |
| 2014 | 936 | 211 (22.5) | 308 (32.9) | 579 (61.9) | 562 (60.0) | 209 (22.3) | 4 (0.4) | 128 (13.7) | 77 (8.2) | 516 (55.1) | 420 (44.9) |
| 2015 | 933 | 207 (22.2) | 332 (35.6) | 602 (64.5) | 568 (60.9) | 187 (20.0) | 8 (0.9) | 129 (13.8) | 50 (5.4) | 539 (57.8) | 452 (48.4) |
| 2016 | 953 | 200 (21.0) | 336 (35.3) | 581 (61.0) | 594 (62.3) | 204 (21.4) | 16 (1.7) | 118 (12.4) | 70 (7.3) | 549 (57.6) | 438 (46.0) |
| 2017 | 913 | 213 (23.3) | 304 (33.3) | 542 (59.4) | 555 (60.8) | 190 (20.8) | 9 (1.0) | 109 (11.9) | 72 (7.9) | 510 (55.9) | 406 (44.5) |
| 2018 | 979 | 247 (25.2) | 327 (33.4) | 579 (59.1) | 576 (58.8) | 201 (20.5) | 8 (0.8) | 121 (12.4) | 72 (7.4) | 531 (54.2) | 431 (44.0) |
| 2019 | 1,054 | 219 (20.8) | 379 (36.0) | 679 (64.4) | 639 (60.6) | 224 (21.3) | 14 (1.3) | 142 (13.5) | 68 (6.5) | 611 (58.0) | 497 (47.2) |
| 2020 | 980 | 199 (20.3) | 299 (30.5) | 651 (66.4) | 563 (57.4) | 237 (24.2) | 20 (2.0) | 162 (16.5) | 55 (5.6) | 544 (55.5) | 453 (46.2) |
| 2021 | 1,101 | 268 (24.3) | 177 (16.1) | 696 (63.2) | 641 (58.2) | 246 (22.3) | 11 (1.0) | 161 (14.6) | 74 (6.7) | 587 (53.3) | 515 (46.8) |

*including unknown. US, United States; y, years.

© 2025 Cao C et al. *JAMA Network Open*.

**eTable 6. Initial treatment by stage, age and rural-urban continuum code.**

| | | | Any Treatment (%) | | | Single Treatment (%) | | | | Multiple Treatments (%) | |
|---|---|---|---|---|---|---|---|---|---|---|---|
| | N | No Treatment *(%) | Surgery | Chemotherapy | Radiation | Only 1 Treatment | Surgery Only | Chemotherapy Only | Radiation Only | More than 1 Treatment | Chemoradio therapy |
| Localized | | | | | | | | | | | |
| 2006 | 801 | 333 (41.6) | 200 (25.0) | 259 (32.3) | 360 (44.9) | 192 (24.0) | 6 (0.7) | 88 (11.0) | 98 (12.2) | 276 (34.5) | 157 (19.6) |
| 2007 | 890 | 365 (41.0) | 238 (26.7) | 300 (33.7) | 390 (43.8) | 212 (23.8) | 19 (2.1) | 95 (10.7) | 98 (11.0) | 313 (35.2) | 184 (20.7) |
| 2008 | 893 | 380 (42.6) | 207 (23.2) | 272 (30.5) | 401 (44.9) | 213 (23.9) | 11 (1.2) | 88 (9.9) | 114 (12.8) | 200 (22.4) | 171 (19.1) |
| 2009 | 893 | 404 (45.2) | 202 (22.6) | 272 (30.5) | 372 (41.7) | 194 (21.7) | 11 (1.2) | 95 (10.6) | 88 (9.9) | 295 (33.0) | 166 (18.6) |
| 2010 | 889 | 392 (44.1) | 219 (24.6) | 267 (30.0) | 385 (43.3) | 200 (22.5) | 14 (1.6) | 86 (9.7) | 100 (11.2) | 297 (33.4) | 169 (19.0) |
| 2011 | 968 | 447 (46.2) | 239 (24.7) | 261 (27.0) | 405 (41.8) | 213 (22.0) | 12 (1.2) | 91 (9.4) | 110 (11.4) | 308 (31.8) | 157 (16.2) |
| 2012 | 949 | 467 (49.2) | 210 (22.1) | 249 (26.2) | 364 (38.4) | 198 (20.9) | 15 (1.6) | 92 (9.7) | 91 (9.6) | 284 (29.9) | 146 (15.4) |
| 2013 | 997 | 484 (48.5) | 223 (22.4) | 269 (27.0) | 398 (39.9) | 208 (20.9) | 11 (1.1) | 93 (9.3) | 104 (10.4) | 305 (30.6) | 165 (16.5) |
| 2014 | 1,051 | 486 (46.2) | 232 (22.1) | 293 (27.9) | 417 (39.7) | 257 (24.5) | 13 (1.2) | 120 (11.4) | 124 (11.8) | 308 (29.3) | 158 (15.0) |
| 2015 | 972 | 465 (47.8) | 207 (21.3) | 268 (27.6) | 364 (37.4) | 234 (24.1) | 13 (1.3) | 114 (11.7) | 107 (11.0) | 273 (28.1) | 138 (14.2) |
| 2016 | 1,040 | 498 (47.9) | 225 (21.6) | 250 (24.0) | 435 (41.8) | 241 (23.2) | 14 (1.3) | 80 (7.7) | 147 (14.1) | 301 (28.9) | 156 (15.0) |
| 2017 | 998 | 489 (49.0) | 170 (17.0) | 271 (27.2) | 383 (38.4) | 251 (25.2) | 14 (1.4) | 102 (10.2) | 135 (13.5) | 258 (25.9) | 159 (15.9) |
| 2018 | 1,095 | 509 (46.5) | 196 (17.9) | 309 (28.2) | 442 (40.4) | 285 (26.0) | 21 (1.9) | 108 (9.9) | 156 (14.2) | 301 (27.5) | 186 (17.0) |
| 2019 | 1,066 | 505 (47.4) | 200 (18.8) | 326 (30.6) | 423 (39.7) | 245 (23.0) | 17 (1.6) | 103 (9.7) | 125 (11.7) | 316 (29.6) | 205 (19.2) |
| 2020 | 997 | 457 (45.8) | 199 (20.0) | 302 (30.3) | 389 (39.0) | 251 (25.2) | 23 (2.3) | 110 (11.0) | 118 (11.8) | 289 (29.0) | 174 (17.5) |
| 2021 | 1,149 | 490 (42.6) | 203 (17.7) | 376 (32.7) | 491 (42.7) | 311 (27.1) | 22 (1.9) | 131 (11.4) | 158 (13.8) | 348 (30.3) | 230 (20.0) |
| | | | | | | | | | | | |

© 2025 Cao C et al. *JAMA Network Open*.

| | | | | Any Treatment (%) | | | Single Treatment (%) | | | Multiple Treatments (%) | |
|---|---|---|---|---|---|---|---|---|---|---|---|
| | N | No Treatment *(%) | Surgery | Chemotherapy | Radiation | Only 1 Treatment | Surgery Only | Chemotherapy Only | Radiation Only | More than 1 Treatment | Chemoradiotherapy |
| Localized, Age<65y | | | | | | | | | | | |
| 2006 | 504 | 200 (39.7) | 149 (29.6) | 165 (32.7) | 239 (47.4) | 112 (22.2) | 5 (1.0) | 51 (10.1) | 56 (11.1) | 192 (38.1) | 105 (20.8) |
| 2007 | 556 | 211 (37.9) | 177 (31.8) | 193 (34.7) | 262 (47.1) | 129 (23.2) | 15 (2.7) | 51 (9.2) | 63 (11.3) | 216 (38.8) | 125 (22.5) |
| 2008 | 523 | 209 (40.0) | 146 (27.9) | 176 (33.7) | 246 (47.0) | 113 (21.6) | 6 (1.1) | 52 (9.9) | 55 (10.5) | 201 (38.4) | 114 (21.8) |
| 2009 | 513 | 213 (41.5) | 141 (27.5) | 168 (32.7) | 234 (45.6) | 105 (20.5) | 9 (1.8) | 51 (9.9) | 45 (8.8) | 195 (38.0) | 111 (21.6) |
| 2010 | 495 | 207 (41.8) | 147 (29.7) | 153 (30.9) | 223 (45.1) | 101 (20.4) | 10 (2.0) | 45 (9.1) | 46 (9.3) | 187 (37.8) | 98 (19.8) |
| 2011 | 563 | 242 (43.0) | 166 (29.5) | 160 (28.4) | 245 (43.5) | 126 (22.4) | 9 (1.6) | 55 (9.8) | 62 (11.0) | 195 (34.6) | 93 (16.5) |
| 2012 | 518 | 243 (46.9) | 139 (26.8) | 147 (28.4) | 210 (40.5) | 96 (18.5) | 9 (1.7) | 48 (9.3) | 39 (7.5) | 179 (34.6) | 91 (17.6) |
| 2013 | 541 | 252 (46.6) | 141 (26.1) | 160 (29.6) | 222 (41.0) | 104 (19.2) | 9 (1.7) | 52 (9.6) | 43 (7.9) | 185 (34.2) | 102 (18.9) |
| 2014 | 544 | 252 (46.3) | 135 (24.8) | 159 (29.2) | 217 (39.9) | 116 (21.3) | 8 (1.5) | 58 (10.7) | 50 (9.2) | 176 (32.4) | 92 (16.9) |
| 2015 | 511 | 249 (48.7) | 120 (23.5) | 146 (28.6) | 183 (35.8) | 111 (21.7) | 6 (1.2) | 60 (11.7) | 45 (8.8) | 151 (29.5) | 73 (14.3) |
| 2016 | 536 | 252 (47.0) | 129 (24.1) | 141 (26.3) | 219 (40.9) | 119 (22.2) | 9 (1.7) | 47 (8.8) | 63 (11.8) | 165 (30.8) | 85 (15.9) |
| 2017 | 523 | 262 (50.1) | 98 (18.7) | 152 (29.1) | 194 (37.1) | 113 (21.6) | 3 (0.6) | 57 (10.9) | 53 (10.1) | 148 (28.3) | 88 (16.8) |
| 2018 | 529 | 232 (43.9) | 119 (22.5) | 169 (31.9) | 218 (41.2) | 126 (23.8) | 13 (2.5) | 55 (10.4) | 58 (11.0) | 171 (32.3) | 103 (19.5) |
| 2019 | 515 | 243 (47.2) | 116 (22.5) | 162 (31.5) | 204 (39.6) | 106 (20.6) | 11 (2.1) | 50 (9.7) | 45 (8.7) | 166 (32.2) | 105 (20.4) |
| 2020 | 493 | 222 (45.0) | 112 (22.7) | 155 (31.4) | 195 (39.6) | 113 (22.9) | 11 (2.2) | 52 (10.5) | 50 (10.1) | 158 (32.0) | 90 (18.3) |
| 2021 | 537 | 228 (42.5) | 111 (20.7) | 182 (33.9) | 225 (41.9) | 137 (25.5) | 12 (2.2) | 63 (11.7) | 62 (11.5) | 172 (32.0) | 110 (20.5) |

© 2025 Cao C et al. *JAMA Network Open*.

| | | | | Any Treatment (%) | | | Single Treatment (%) | | | Multiple Treatments (%) | |
|---|---|---|---|---|---|---|---|---|---|---|---|
| | N | No Treatment *(%) | Surgery | Chemotherapy | Radiation | Only 1 Treatment | Surgery Only | Chemotherapy Only | Radiation Only | More than 1 Treatment | Chemoradiotherapy |
| Localized, Age≥65y | | | | | | | | | | | |
| 2006 | 297 | 133 (44.8) | 51 (17.2) | 94 (31.6) | 121 (40.7) | 80 (26.9) | 1 (0.3) | 37 (12.5) | 42 (14.1) | 84 (28.3) | 52 (17.5) |
| 2007 | 334 | 154 (46.1) | 61 (18.3) | 107 (32.0) | 128 (38.3) | 83 (24.9) | 4 (1.2) | 44 (13.2) | 35 (10.5) | 97 (29.0) | 59 (17.7) |
| 2008 | 370 | 171 (46.2) | 61 (16.5) | 96 (25.9) | 155 (41.9) | 100 (27.0) | 5 (1.4) | 36 (9.7) | 59 (15.9) | 99 (26.8) | 57 (15.4) |
| 2009 | 380 | 191 (50.3) | 61 (16.1) | 104 (27.4) | 138 (36.3) | 89 (23.4) | 2 (0.5) | 44 (11.6) | 43 (11.3) | 100 (26.3) | 55 (14.5) |
| 2010 | 394 | 185 (47.0) | 72 (18.3) | 114 (28.9) | 162 (41.1) | 99 (25.1) | 4 (1.0) | 41 (10.4) | 54 (13.7) | 110 (27.9) | 71 (18.0) |
| 2011 | 405 | 205 (50.6) | 73 (18.0) | 101 (24.9) | 160 (39.5) | 87 (21.5) | 3 (0.7) | 36 (8.9) | 48 (11.9) | 113 (27.9) | 64 (15.8) |
| 2012 | 431 | 224 (52.0) | 71 (16.5) | 102 (23.7) | 154 (35.7) | 102 (23.7) | 6 (1.4) | 44 (10.2) | 52 (12.1) | 105 (24.4) | 55 (12.8) |
| 2013 | 456 | 232 (50.9) | 82 (18.0) | 109 (23.9) | 176 (38.6) | 104 (22.8) | 2 (0.4) | 41 (9.0) | 61 (13.4) | 120 (26.3) | 63 (13.8) |
| 2014 | 507 | 234 (46.2) | 97 (19.1) | 134 (26.4) | 200 (39.4) | 141 (27.8) | 5 (1.0) | 62 (12.2) | 74 (14.6) | 132 (26.0) | 66 (13.0) |
| 2015 | 461 | 216 (46.9) | 87 (18.9) | 122 (26.5) | 181 (39.3) | 123 (26.7) | 7 (1.5) | 54 (11.7) | 62 (13.4) | 122 (26.5) | 65 (14.1) |
| 2016 | 504 | 246 (48.8) | 96 (19.0) | 109 (21.6) | 215 (42.7) | 122 (24.2) | 5 (1.0) | 33 (6.5) | 84 (16.7) | 136 (27.0) | 71 (14.1) |
| 2017 | 475 | 227 (47.8) | 72 (15.2) | 119 (25.1) | 189 (39.8) | 138 (29.1) | 11 (2.3) | 45 (9.5) | 82 (17.3) | 110 (23.2) | 71 (14.9) |
| 2018 | 566 | 277 (48.9) | 77 (13.6) | 140 (24.7) | 224 (39.6) | 159 (28.1) | 8 (1.4) | 53 (9.4) | 98 (17.3) | 130 (23.0) | 83 (14.7) |
| 2019 | 551 | 262 (47.5) | 84 (15.2) | 164 (29.8) | 219 (39.7) | 139 (25.2) | 6 (1.1) | 53 (9.6) | 80 (14.5) | 150 (27.2) | 100 (18.1) |
| 2020 | 504 | 235 (46.6) | 87 (17.3) | 147 (29.2) | 194 (38.5) | 138 (27.4) | 12 (2.4) | 58 (11.5) | 68 (13.5) | 131 (26.0) | 84 (16.7) |
| 2021 | 612 | 262 (42.8) | 92 (15.0) | 194 (31.7) | 266 (43.5) | 174 (28.4) | 10 (1.6) | 68 (11.1) | 96 (15.7) | 176 (28.8) | 120 (19.6) |
| | | | | | | | | | | | |

© 2025 Cao C et al. *JAMA Network Open*.

| | | | | Any Treatment (%) | | | Single Treatment (%) | | | Multiple Treatments (%) | |
|---|---|---|---|---|---|---|---|---|---|---|---|
| | N | No Treatment *(%) | Surgery | Chemotherapy | Radiation | Only 1 Treatment | Surgery Only | Chemotherapy Only | Radiation Only | More than 1 Treatment | Chemoradiotherapy |
| Localized, Metropolitan | | | | | | | | | | | |
| 2006 | 670 | 280 (41.8) | 163 (24.3) | 217 (32.4) | 304 (45.4) | 159 (23.7) | 6 (0.9) | 72 (10.7) | 81 (12.1) | 231 (34.5) | 137 (20.4) |
| 2007 | 733 | 295 (40.2) | 193 (26.3) | 250 (34.1) | 317 (43.2) | 187 (25.5) | 17 (2.3) | 85 (11.6) | 85 (11.6) | 251 (34.2) | 146 (19.9) |
| 2008 | 758 | 322 (42.5) | 182 (24.0) | 231 (30.5) | 349 (46.0) | 171 (22.6) | 11 (1.5) | 65 (8.6) | 95 (12.5) | 265 (35.0) | 155 (20.4) |
| 2009 | 767 | 354 (46.2) | 174 (22.7) | 224 (29.2) | 319 (41.6) | 160 (20.9) | 9 (1.2) | 77 (10.0) | 74 (9.6) | 253 (33.0) | 139 (18.1) |
| 2010 | 768 | 340 (44.3) | 188 (24.5) | 227 (29.6) | 332 (43.2) | 176 (22.9) | 13 (1.7) | 76 (9.9) | 87 (11.3) | 252 (32.8) | 144 (18.8) |
| 2011 | 835 | 382 (45.7) | 209 (25.0) | 222 (26.6) | 358 (42.9) | 184 (22.0) | 10 (1.2) | 73 (8.7) | 101 (12.1) | 269 (32.2) | 137 (16.4) |
| 2012 | 804 | 397 (49.4) | 182 (22.6) | 211 (26.2) | 307 (38.2) | 166 (20.6) | 15 (1.9) | 77 (9.6) | 74 (9.2) | 241 (30.0) | 126 (15.7) |
| 2013 | 846 | 401 (47.4) | 196 (23.2) | 230 (27.2) | 347 (41.0) | 178 (21.0) | 9 (1.1) | 81 (9.6) | 88 (10.4) | 267 (31.6) | 141 (16.7) |
| 2014 | 900 | 413 (45.9) | 203 (22.6) | 258 (28.7) | 354 (39.3) | 222 (24.7) | 13 (1.4) | 107 (11.9) | 102 (11.3) | 265 (29.4) | 138 (15.3) |
| 2015 | 841 | 405 (48.2) | 179 (21.3) | 227 (27.0) | 315 (37.5) | 203 (24.1) | 10 (1.2) | 97 (11.5) | 96 (11.4) | 233 (27.7) | 116 (13.8) |
| 2016 | 889 | 427 (48.0) | 194 (21.8) | 212 (23.8) | 376 (42.3) | 199 (22.4) | 11 (1.2) | 64 (7.2) | 124 (13.9) | 263 (29.6) | 137 (15.4) |
| 2017 | 871 | 427 (49.0) | 149 (17.1) | 239 (27.4) | 330 (37.9) | 221 (25.4) | 13 (1.5) | 92 (10.6) | 116 (13.3) | 223 (25.6) | 138 (15.8) |
| 2018 | 938 | 431 (45.9) | 169 (18.0) | 274 (29.2) | 379 (40.4) | 244 (26.0) | 20 (2.1) | 93 (9.9) | 131 (14.0) | 263 (28.0) | 166 (17.7) |
| 2019 | 905 | 429 (47.4) | 171 (18.9) | 279 (30.8) | 360 (39.8) | 201 (22.2) | 12 (1.3) | 89 (9.8) | 100 (11.0) | 275 (30.4) | 175 (19.3) |
| 2020 | 850 | 397 (46.7) | 167 (19.6) | 250 (29.4) | 331 (38.9) | 210 (24.7) | 15 (1.8) | 92 (10.8) | 103 (12.1) | 243 (28.6) | 143 (16.8) |
| 2021 | 957 | 392 (41.0) | 176 (18.4) | 319 (33.3) | 417 (43.6) | 273 (28.5) | 21 (2.2) | 116 (12.1) | 136 (14.2) | 292 (30.5) | 192 (20.1) |

© 2025 Cao C et al. *JAMA Network Open*.

| | | | | Any Treatment (%) | | | Single Treatment (%) | | | Multiple Treatments (%) | |
|---|---|---|---|---|---|---|---|---|---|---|---|
| | N | No Treatment *(%) | Surgery | Chemotherapy | Radiation | Only 1 Treatment | Surgery Only | Chemotherapy Only | Radiation Only | More than 1 Treatment | Chemoradiotherapy |
| Localized, Nonmetropolitan | | | | | | | | | | | |
| 2006 | 129 | 51 (39.5) | 37 (28.7) | 42 (32.6) | 56 (43.4) | 33 (25.6) | 0 (0.0) | 16 (12.4) | 17 (13.2) | 45 (34.9) | 20 (15.5) |
| 2007 | 157 | 70 (44.6) | 45 (28.7) | 50 (31.8) | 73 (46.5) | 25 (15.9) | 2 (1.3) | 10 (6.4) | 13 (8.3) | 62 (39.5) | 38 (24.2) |
| 2008 | 135 | 58 (43.0) | 25 (18.5) | 41 (30.4) | 52 (38.5) | 42 (31.1) | 0 (0.0) | 23 (17.0) | 19 (14.1) | 35 (25.9) | 16 (11.9) |
| 2009 | 126 | 50 (39.7) | 28 (22.2) | 48 (38.1) | 53 (42.1) | 34 (27.0) | 2 (1.6) | 18 (14.3) | 14 (11.1) | 42 (33.3) | 27 (21.4) |
| 2010 | 121 | 52 (43.0) | 31 (25.6) | 40 (33.1) | 53 (43.8) | 24 (19.8) | 1 (0.8) | 10 (8.3) | 13 (10.7) | 45 (37.2) | 25 (20.7) |
| 2011 | 133 | 65 (48.9) | 30 (22.6) | 39 (29.3) | 47 (35.3) | 29 (21.8) | 2 (1.5) | 18 (13.5) | 9 (6.8) | 39 (29.3) | 20 (15.0) |
| 2012 | 144 | 70 (48.6) | 27 (18.8) | 37 (25.7) | 56 (38.9) | 32 (22.2) | 0 (0.0) | 15 (10.4) | 17 (11.8) | 42 (29.2) | 19 (13.2) |
| 2013 | 151 | 83 (55.0) | 27 (17.9) | 39 (25.8) | 51 (33.8) | 30 (19.9) | 2 (1.3) | 12 (7.9) | 16 (10.6) | 38 (25.2) | 24 (15.9) |
| 2014 | 151 | 73 (48.3) | 29 (19.2) | 35 (23.2) | 63 (41.7) | 35 (23.2) | 0 (0.0) | 13 (8.6) | 22 (14.6) | 43 (28.5) | 20 (13.2) |
| 2015 | 131 | 60 (45.8) | 28 (21.4) | 41 (31.3) | 49 (37.4) | 31 (23.7) | 3 (2.3) | 17 (13.0) | 11 (8.4) | 40 (30.5) | 22 (16.8) |
| 2016 | 150 | 71 (47.3) | 31 (20.7) | 38 (25.3) | 57 (38.0) | 41 (27.3) | 3 (2.0) | 16 (10.7) | 22 (14.7) | 38 (25.3) | 19 (12.7) |
| 2017 | 124 | 61 (49.2) | 20 (16.1) | 31 (25.0) | 52 (41.9) | 29 (23.4) | 1 (0.8) | 9 (7.3) | 19 (15.3) | 34 (27.4) | 21 (16.9) |
| 2018 | 157 | 78 (49.7) | 27 (17.2) | 35 (22.3) | 63 (40.1) | 41 (26.1) | 1 (0.6) | 15 (9.6) | 25 (15.9) | 38 (24.2) | 20 (12.7) |
| 2019 | 161 | 76 (47.2) | 29 (18.0) | 47 (29.2) | 63 (39.1) | 44 (27.3) | 5 (3.1) | 14 (8.7) | 25 (15.5) | 41 (25.5) | 30 (18.6) |
| 2020 | 146 | 60 (41.1) | 31 (21.2) | 51 (34.9) | 57 (39.0) | 41 (28.1) | 8 (5.5) | 18 (12.3) | 15 (10.3) | 45 (30.8) | 30 (20.5) |
| 2021 | 191 | 97 (50.8) | 27 (14.1) | 57 (29.8) | 74 (38.7) | 38 (19.9) | 1 (0.5) | 15 (7.9) | 22 (11.5) | 56 (29.3) | 38 (19.9) |
| | | | | | | | | | | | |

© 2025 Cao C et al. *JAMA Network Open*.

| | | | | Any Treatment (%) | | | Single Treatment (%) | | | Multiple Treatments (%) | |
|---|---|---|---|---|---|---|---|---|---|---|---|
| | N | No Treatment *(%) | Surgery | Chemotherapy | Radiation | Only 1 Treatment | Surgery Only | Chemotherapy Only | Radiation Only | More than 1 Treatment | Chemoradiotherapy |
| Regional | | | | | | | | | | | |
| 2006 | 2,856 | 432 (15.1) | 1,132 (39.6) | 2,015 (70.6) | 1,963 (68.7) | 526 (18.4) | 32 (1.1) | 324 (11.3) | 170 (6.0) | 1,898 (66.5) | 1,586 (55.5) |
| 2007 | 3,117 | 430 (13.8) | 1,229 (39.4) | 2,294 (73.6) | 2,179 (69.9) | 555 (17.8) | 27 (0.9) | 370 (11.9) | 158 (5.1) | 2,132 (68.4) | 1,813 (58.2) |
| 2008 | 3,287 | 430 (13.1) | 1,270 (38.6) | 2,464 (75.0) | 2,260 (68.8) | 636 (19.3) | 26 (0.8) | 457 (13.9) | 153 (4.7) | 2,221 (67.6) | 1,893 (57.6) |
| 2009 | 3,542 | 478 (13.5) | 1,355 (38.3) | 2,676 (75.6) | 2,484 (70.1) | 639 (18.0) | 31 (0.9) | 462 (13.0) | 146 (4.1) | 2,425 (68.5) | 2,127 (60.1) |
| 2010 | 3,520 | 441 (12.5) | 1,475 (41.9) | 2,675 (76.0) | 2,513 (71.4) | 575 (16.3) | 31 (0.9) | 414 (11.8) | 130 (3.7) | 2,504 (71.1) | 2,140 (60.8) |
| 2011 | 3,709 | 490 (13.2) | 1,602 (43.2) | 2,785 (75.1) | 2,614 (70.5) | 608 (16.4) | 28 (0.8) | 437 (11.8) | 143 (3.9) | 2,611 (70.4) | 2,208 (59.5) |
| 2012 | 3,776 | 473 (12.5) | 1,613 (42.7) | 2,825 (74.8) | 2,694 (71.3) | 631 (16.7) | 30 (0.8) | 453 (12.0) | 148 (3.9) | 2,672 (70.8) | 2,246 (59.5) |
| 2013 | 3,991 | 531 (13.3) | 1,657 (41.5) | 2,884 (72.3) | 2,854 (71.5) | 689 (17.3) | 30 (0.8) | 441 (11.0) | 218 (5.5) | 2,771 (69.4) | 2,308 (57.8) |
| 2014 | 4,253 | 581 (13.7) | 1,731 (40.7) | 2,994 (70.4) | 3,027 (71.2) | 767 (18.0) | 30 (0.7) | 476 (11.2) | 261 (6.1) | 2,905 (68.3) | 2,379 (55.9) |
| 2015 | 4,480 | 645 (14.4) | 1,870 (41.7) | 3,110 (69.4) | 3,189 (71.2) | 760 (17.0) | 53 (1.2) | 465 (10.4) | 242 (5.4) | 3,075 (68.6) | 2,517 (56.2) |
| 2016 | 4,845 | 681 (14.1) | 1,945 (40.1) | 3,323 (68.6) | 3,474 (71.7) | 821 (16.9) | 52 (1.1) | 472 (9.7) | 297 (6.1) | 3,343 (69.0) | 2,685 (55.4) |
| 2017 | 4,904 | 721 (14.7) | 1,905 (38.8) | 3,340 (68.1) | 3,453 (70.4) | 844 (17.2) | 65 (1.3) | 489 (10.0) | 290 (5.9) | 3,339 (68.1) | 2,675 (54.5) |
| 2018 | 5,112 | 751 (14.7) | 2,034 (39.8) | 3,509 (68.6) | 3,591 (70.2) | 899 (17.6) | 63 (1.2) | 527 (10.3) | 309 (6.0) | 3,462 (67.7) | 2,802 (54.8) |
| 2019 | 5,401 | 769 (14.2) | 2,242 (41.5) | 3,864 (71.5) | 3,767 (69.7) | 886 (16.4) | 65 (1.2) | 575 (10.6) | 246 (4.6) | 3,746 (69.4) | 3,064 (56.7) |
| 2020 | 5,169 | 623 (12.1) | 2,057 (39.8) | 3,793 (73.4) | 3,570 (69.1) | 954 (18.5) | 80 (1.5) | 635 (12.3) | 239 (4.6) | 3,592 (69.5) | 2,897 (56.0) |
| 2021 | 5,286 | 764 (14.5) | 1,182 (22.4) | 3,816 (72.2) | 3,632 (68.7) | 1,043 (19.7) | 60 (1.1) | 690 (13.1) | 293 (5.5) | 3,479 (65.8) | 2,986 (56.5) |
| | | | | | | | | | | | |

© 2025 Cao C et al. *JAMA Network Open*.

| | | | | Any Treatment (%) | | | Single Treatment (%) | | | Multiple Treatments (%) | |
|---|---|---|---|---|---|---|---|---|---|---|---|
| | N | No Treatment *(%) | Surgery | Chemotherapy | Radiation | Only 1 Treatment | Surgery Only | Chemotherapy Only | Radiation Only | More than 1 Treatment | Chemoradiotherapy |
| Regional, Age<65y | | | | | | | | | | | |
| 2006 | 2,039 | 267 (13.1) | 898 (44.0) | 1,508 (74.0) | 1,422 (69.7) | 352 (17.3) | 28 (1.4) | 238 (11.7) | 86 (4.2) | 1,420 (69.6) | 1,186 (58.2) |
| 2007 | 2,214 | 273 (12.3) | 940 (42.5) | 1,688 (76.2) | 1,570 (70.9) | 367 (16.6) | 22 (1.0) | 264 (11.9) | 81 (3.7) | 1,574 (71.1) | 1,339 (60.5) |
| 2008 | 2,306 | 266 (11.5) | 1,007 (43.7) | 1,788 (77.5) | 1,616 (70.1) | 403 (17.5) | 17 (0.7) | 316 (13.7) | 70 (3.0) | 1,637 (71.0) | 1,381 (59.9) |
| 2009 | 2,518 | 279 (11.1) | 1,063 (42.2) | 1,992 (79.1) | 1,822 (72.4) | 419 (16.6) | 24 (1.0) | 319 (12.7) | 76 (3.0) | 1,820 (72.3) | 1,599 (63.5) |
| 2010 | 2,460 | 268 (10.9) | 1,137 (46.2) | 1,926 (78.3) | 1,800 (73.2) | 362 (14.7) | 26 (1.1) | 269 (10.9) | 67 (2.7) | 1,830 (74.4) | 1,560 (63.4) |
| 2011 | 2,591 | 312 (12.0) | 1,229 (47.4) | 2,009 (77.5) | 1,855 (71.6) | 376 (14.5) | 18 (0.7) | 291 (11.2) | 67 (2.6) | 1,903 (73.4) | 1,603 (61.9) |
| 2012 | 2,515 | 274 (10.9) | 1,178 (46.8) | 1,957 (77.8) | 1,822 (72.4) | 391 (15.5) | 17 (0.7) | 306 (12.2) | 68 (2.7) | 1,850 (73.6) | 1,555 (61.8) |
| 2013 | 2,634 | 284 (10.8) | 1,225 (46.5) | 2,003 (76.0) | 1,936 (73.5) | 413 (15.7) | 22 (0.8) | 288 (10.9) | 103 (3.9) | 1,937 (73.5) | 1,611 (61.2) |
| 2014 | 2,709 | 311 (11.5) | 1,200 (44.3) | 2,036 (75.2) | 1,968 (72.6) | 434 (16.0) | 17 (0.6) | 305 (11.3) | 112 (4.1) | 1,964 (72.5) | 1,623 (59.9) |
| 2015 | 2,836 | 353 (12.4) | 1,318 (46.5) | 2,069 (73.0) | 2,076 (73.2) | 411 (14.5) | 33 (1.2) | 279 (9.8) | 99 (3.5) | 2,072 (73.1) | 1,695 (59.8) |
| 2016 | 2,955 | 352 (11.9) | 1,312 (44.4) | 2,131 (72.1) | 2,135 (72.3) | 466 (15.8) | 46 (1.6) | 305 (10.3) | 115 (3.9) | 2,137 (72.3) | 1,709 (57.8) |
| 2017 | 2,992 | 379 (12.7) | 1,287 (43.0) | 2,168 (72.5) | 2,152 (71.9) | 441 (14.7) | 39 (1.3) | 294 (9.8) | 108 (3.6) | 2,172 (72.6) | 1,746 (58.4) |
| 2018 | 2,965 | 392 (13.2) | 1,303 (43.9) | 2,146 (72.4) | 2,093 (70.6) | 467 (15.8) | 48 (1.6) | 322 (10.9) | 97 (3.3) | 2,106 (71.0) | 1,714 (57.8) |
| 2019 | 3,183 | 411 (12.9) | 1,429 (44.9) | 2,389 (75.1) | 2,275 (71.5) | 425 (13.4) | 35 (1.1) | 310 (9.7) | 80 (2.5) | 2,347 (73.7) | 1,927 (60.5) |
| 2020 | 2,903 | 296 (10.2) | 1,240 (42.7) | 2,235 (77.0) | 2,053 (70.7) | 485 (16.7) | 39 (1.3) | 367 (12.6) | 79 (2.7) | 2,122 (73.1) | 1,720 (59.2) |
| 2021 | 2,917 | 376 (12.9) | 747 (25.6) | 2,190 (75.1) | 2,021 (69.3) | 543 (18.6) | 35 (1.2) | 394 (13.5) | 114 (3.9) | 1,998 (68.5) | 1,705 (58.5) |
| | | | | | | | | | | | |

© 2025 Cao C et al. *JAMA Network Open*.

| | | | | Any Treatment (%) | | | Single Treatment (%) | | | Multiple Treatments (%) | |
|---|---|---|---|---|---|---|---|---|---|---|---|
| | N | No Treatment *(%) | Surgery | Chemotherapy | Radiation | Only 1 Treatment | Surgery Only | Chemotherapy Only | Radiation Only | More than 1 Treatment | Chemoradiotherapy |
| Regional, Age≥65y | | | | | | | | | | | |
| 2006 | 817 | 165 (20.2) | 234 (28.6) | 507 (62.1) | 541 (66.2) | 174 (21.3) | 4 (0.5) | 86 (10.5) | 84 (10.3) | 478 (58.5) | 400 (49.0) |
| 2007 | 903 | 157 (17.4) | 289 (32.0) | 606 (67.1) | 609 (67.4) | 188 (20.8) | 5 (0.6) | 106 (11.7) | 77 (8.5) | 558 (61.8) | 474 (52.5) |
| 2008 | 981 | 164 (16.7) | 263 (26.8) | 676 (68.9) | 644 (65.6) | 233 (23.8) | 9 (0.9) | 141 (14.4) | 83 (8.5) | 584 (59.5) | 512 (52.2) |
| 2009 | 1,024 | 199 (19.4) | 292 (28.5) | 684 (66.8) | 662 (64.6) | 220 (21.5) | 7 (0.7) | 143 (14.0) | 70 (6.8) | 605 (59.1) | 528 (51.6) |
| 2010 | 1,060 | 173 (16.3) | 338 (31.9) | 749 (70.7) | 713 (67.3) | 213 (20.1) | 5 (0.5) | 145 (13.7) | 63 (5.9) | 674 (63.6) | 580 (54.7) |
| 2011 | 1,118 | 178 (15.9) | 373 (33.4) | 776 (69.4) | 759 (67.9) | 232 (20.8) | 10 (0.9) | 146 (13.1) | 76 (6.8) | 708 (63.3) | 605 (54.1) |
| 2012 | 1,261 | 199 (15.8) | 435 (34.5) | 868 (68.8) | 872 (69.2) | 240 (19.0) | 13 (1.0) | 147 (11.7) | 80 (6.3) | 822 (65.2) | 691 (54.8) |
| 2013 | 1,357 | 247 (18.2) | 432 (31.8) | 881 (64.9) | 918 (67.6) | 276 (20.3) | 8 (0.6) | 153 (11.3) | 115 (8.5) | 834 (61.5) | 697 (51.4) |
| 2014 | 1,544 | 270 (17.5) | 531 (34.4) | 958 (62.0) | 1,059 (68.6) | 333 (21.6) | 13 (0.8) | 171 (11.1) | 149 (9.7) | 941 (60.9) | 756 (49.0) |
| 2015 | 1,644 | 292 (17.8) | 552 (33.6) | 1,041 (63.3) | 1,113 (67.7) | 349 (21.2) | 20 (1.2) | 186 (11.3) | 143 (8.7) | 1,003 (61.0) | 822 (50.0) |
| 2016 | 1,890 | 329 (17.4) | 633 (33.5) | 1,192 (63.1) | 1,339 (70.8) | 355 (18.8) | 6 (0.3) | 167 (8.8) | 182 (9.6) | 1,206 (63.8) | 976 (51.6) |
| 2017 | 1,912 | 342 (17.9) | 618 (32.3) | 1,172 (61.3) | 1,301 (68.0) | 403 (21.1) | 26 (1.4) | 195 (10.2) | 182 (9.5) | 1,167 (61.0) | 929 (48.6) |
| 2018 | 2,147 | 359 (16.7) | 731 (34.0) | 1,363 (63.5) | 1,498 (69.8) | 432 (20.1) | 15 (0.7) | 205 (9.5) | 212 (9.9) | 1,356 (63.2) | 1,088 (50.7) |
| 2019 | 2,218 | 358 (16.1) | 813 (36.7) | 1,475 (66.5) | 1,492 (67.3) | 461 (20.8) | 30 (1.4) | 265 (11.9) | 166 (7.5) | 1,399 (63.1) | 1,137 (51.3) |
| 2020 | 2,266 | 327 (14.4) | 817 (36.1) | 1,558 (68.8) | 1,517 (66.9) | 469 (20.7) | 41 (1.8) | 268 (11.8) | 160 (7.1) | 1,470 (64.9) | 1,177 (51.9) |
| 2021 | 2,369 | 388 (16.4) | 435 (18.4) | 1,626 (68.6) | 1,611 (68.0) | 500 (21.1) | 25 (1.1) | 296 (12.5) | 179 (7.6) | 1,481 (62.5) | 1,281 (54.1) |
| | | | | | | | | | | | |

© 2025 Cao C et al. *JAMA Network Open*.

| | | | | Any Treatment (%) | | | Single Treatment (%) | | | Multiple Treatments (%) | |
|---|---|---|---|---|---|---|---|---|---|---|---|
| | N | No Treatment *(%) | Surgery | Chemotherapy | Radiation | Only 1 Treatment | Surgery Only | Chemotherapy Only | Radiation Only | More than 1 Treatment | Chemoradiotherapy |
| Regional, Metropolitan | | | | | | | | | | | |
| 2006 | 2,482 | 376 (15.1) | 973 (39.2) | 1,756 (70.7) | 1,702 (68.6) | 451 (18.2) | 29 (1.2) | 283 (11.4) | 139 (5.6) | 1,655 (66.7) | 1,381 (55.6) |
| 2007 | 2,696 | 365 (13.5) | 1,057 (39.2) | 2,003 (74.3) | 1,893 (70.2) | 478 (17.7) | 23 (0.9) | 322 (11.9) | 133 (4.9) | 1,853 (68.7) | 1,588 (58.9) |
| 2008 | 2,883 | 368 (12.8) | 1,119 (38.8) | 2,188 (75.9) | 1,999 (69.3) | 544 (18.9) | 23 (0.8) | 392 (13.6) | 129 (4.5) | 1,971 (68.4) | 1,695 (58.8) |
| 2009 | 3,104 | 417 (13.4) | 1,181 (38.0) | 2,352 (75.8) | 2,180 (70.2) | 556 (17.9) | 26 (0.8) | 403 (13.0) | 127 (4.1) | 2,131 (68.7) | 1,871 (60.3) |
| 2010 | 3,089 | 390 (12.6) | 1,296 (42.0) | 2,349 (76.0) | 2,208 (71.5) | 492 (15.9) | 27 (0.9) | 354 (11.5) | 111 (3.6) | 2,207 (71.4) | 1,885 (61.0) |
| 2011 | 3,220 | 419 (13.0) | 1,401 (43.5) | 2,421 (75.2) | 2,272 (70.6) | 536 (16.6) | 26 (0.8) | 380 (11.8) | 130 (4.0) | 2,265 (70.3) | 1,918 (59.6) |
| 2012 | 3,291 | 410 (12.5) | 1,382 (42.0) | 2,467 (75.0) | 2,341 (71.1) | 568 (17.3) | 24 (0.7) | 413 (12.5) | 131 (4.0) | 2,313 (70.3) | 1,951 (59.3) |
| 2013 | 3,447 | 439 (12.7) | 1,451 (42.1) | 2,505 (72.7) | 2,501 (72.6) | 585 (17.0) | 25 (0.7) | 367 (10.6) | 193 (5.6) | 2,423 (70.3) | 2,023 (58.7) |
| 2014 | 3,674 | 490 (13.3) | 1,514 (41.2) | 2,586 (70.4) | 2,646 (72.0) | 642 (17.5) | 28 (0.8) | 393 (10.7) | 221 (6.0) | 2,542 (69.2) | 2,076 (56.5) |
| 2015 | 3,895 | 557 (14.3) | 1,621 (41.6) | 2,694 (69.2) | 2,783 (71.5) | 656 (16.8) | 48 (1.2) | 394 (10.1) | 214 (5.5) | 2,682 (68.9) | 2,187 (56.1) |
| 2016 | 4,220 | 603 (14.3) | 1,676 (39.7) | 2,884 (68.3) | 3,027 (71.7) | 705 (16.7) | 41 (1.0) | 403 (9.5) | 261 (6.2) | 2,912 (69.0) | 2,335 (55.3) |
| 2017 | 4,288 | 624 (14.6) | 1,664 (38.8) | 2,927 (68.3) | 3,040 (70.9) | 720 (16.8) | 57 (1.3) | 416 (9.7) | 247 (5.8) | 2,944 (68.7) | 2,360 (55.0) |
| 2018 | 4,438 | 624 (14.1) | 1,765 (39.8) | 3,061 (69.0) | 3,149 (71.0) | 779 (17.6) | 56 (1.3) | 453 (10.2) | 270 (6.1) | 3,035 (68.4) | 2,452 (55.3) |
| 2019 | 4,636 | 664 (14.3) | 1,917 (41.4) | 3,313 (71.5) | 3,247 (70.0) | 741 (16.0) | 56 (1.2) | 479 (10.3) | 206 (4.4) | 3,231 (69.7) | 2,644 (57.0) |
| 2020 | 4,487 | 535 (11.9) | 1,810 (40.3) | 3,288 (73.3) | 3,122 (69.6) | 801 (17.9) | 68 (1.5) | 530 (11.8) | 203 (4.5) | 3,151 (70.2) | 2,526 (56.3) |
| 2021 | 4,543 | 645 (14.2) | 1,048 (23.1) | 3,278 (72.2) | 3,136 (69.0) | 888 (19.5) | 51 (1.1) | 585 (12.9) | 252 (5.5) | 3,010 (66.3) | 2,567 (56.5) |
| | | | | | | | | | | | |

© 2025 Cao C et al. *JAMA Network Open*.

| | | | | Any Treatment (%) | | | Single Treatment (%) | | | Multiple Treatments (%) | |
|---|---|---|---|---|---|---|---|---|---|---|---|
| | N | No Treatment *(%) | Surgery | Chemotherapy | Radiation | Only 1 Treatment | Surgery Only | Chemotherapy Only | Radiation Only | More than 1 Treatment | Chemoradiotherapy |
| Regional, Nonmetropolitan | | | | | | | | | | | |
| 2006 | 373 | 56 (15.0) | 159 (42.6) | 258 (69.2) | 260 (69.7) | 75 (20.1) | 3 (0.8) | 41 (11.0) | 31 (8.3) | 242 (64.9) | 204 (54.7) |
| 2007 | 417 | 65 (15.6) | 171 (41.0) | 288 (69.1) | 283 (67.9) | 76 (18.2) | 4 (1.0) | 47 (11.3) | 25 (6.0) | 276 (66.2) | 223 (53.5) |
| 2008 | 404 | 62 (15.3) | 151 (37.4) | 276 (68.3) | 261 (64.6) | 92 (22.8) | 3 (0.7) | 65 (16.1) | 24 (5.9) | 250 (61.9) | 198 (49.0) |
| 2009 | 437 | 61 (14.0) | 173 (39.6) | 324 (74.1) | 303 (69.3) | 83 (19.0) | 5 (1.1) | 59 (13.5) | 19 (4.3) | 293 (67.0) | 256 (58.6) |
| 2010 | 426 | 51 (12.0) | 177 (41.5) | 323 (75.8) | 300 (70.4) | 81 (19.0) | 4 (0.9) | 60 (14.1) | 17 (4.0) | 294 (69.0) | 252 (59.2) |
| 2011 | 487 | 71 (14.6) | 200 (41.1) | 363 (74.5) | 340 (69.8) | 72 (14.8) | 2 (0.4) | 57 (11.7) | 13 (2.7) | 344 (70.6) | 289 (59.3) |
| 2012 | 481 | 62 (12.9) | 230 (47.8) | 356 (74.0) | 350 (72.8) | 62 (12.9) | 6 (1.2) | 40 (8.3) | 16 (3.3) | 357 (74.2) | 293 (60.9) |
| 2013 | 541 | 91 (16.8) | 205 (37.9) | 379 (70.1) | 351 (64.9) | 103 (19.0) | 5 (0.9) | 74 (13.7) | 24 (4.4) | 347 (64.1) | 285 (52.7) |
| 2014 | 576 | 91 (15.8) | 217 (37.7) | 406 (70.5) | 378 (65.6) | 124 (21.5) | 2 (0.3) | 83 (14.4) | 39 (6.8) | 361 (62.7) | 301 (52.3) |
| 2015 | 583 | 87 (14.9) | 248 (42.5) | 415 (71.2) | 405 (69.5) | 104 (17.8) | 5 (0.9) | 71 (12.2) | 28 (4.8) | 392 (67.2) | 329 (56.4) |
| 2016 | 624 | 78 (12.5) | 269 (43.1) | 438 (70.2) | 446 (71.5) | 116 (18.6) | 11 (1.8) | 69 (11.1) | 36 (5.8) | 430 (68.9) | 349 (55.9) |
| 2017 | 616 | 97 (15.7) | 241 (39.1) | 413 (67.0) | 413 (67.0) | 124 (20.1) | 8 (1.3) | 73 (11.9) | 43 (7.0) | 395 (64.1) | 315 (51.1) |
| 2018 | 668 | 126 (18.9) | 268 (40.1) | 447 (66.9) | 437 (65.4) | 117 (17.5) | 7 (1.0) | 74 (11.1) | 36 (5.4) | 425 (63.6) | 349 (52.2) |
| 2019 | 761 | 104 (13.7) | 325 (42.7) | 550 (72.3) | 517 (67.9) | 143 (18.8) | 9 (1.2) | 96 (12.6) | 38 (5.0) | 514 (67.5) | 419 (55.1) |
| 2020 | 681 | 88 (12.9) | 247 (36.3) | 505 (74.2) | 447 (65.6) | 152 (22.3) | 12 (1.8) | 105 (15.4) | 35 (5.1) | 441 (64.8) | 371 (54.5) |
| 2021 | 741 | 119 (16.1) | 133 (17.9) | 537 (72.5) | 494 (66.7) | 155 (20.9) | 9 (1.2) | 105 (14.2) | 41 (5.5) | 467 (63.0) | 418 (56.4) |
| | | | | | | | | | | | |

© 2025 Cao C et al. *JAMA Network Open*.

| | | | | Any Treatment (%) | | | Single Treatment (%) | | | Multiple Treatments (%) | |
|---|---|---|---|---|---|---|---|---|---|---|---|
| | N | No Treatment *(%) | Surgery | Chemotherapy | Radiation | Only 1 Treatment | Surgery Only | Chemotherapy Only | Radiation Only | More than 1 Treatment | Chemoradiotherapy |
| Distant | | | | | | | | | | | |
| 2006 | 836 | 149 (17.8) | 233 (27.9) | 588 (70.3) | 524 (62.7) | 191 (22.8) | 3 (0.4) | 138 (16.5) | 50 (6.0) | 496 (59.3) | 428 (51.2) |
| 2007 | 943 | 191 (20.3) | 256 (27.1) | 678 (71.9) | 564 (59.8) | 202 (21.4) | 4 (0.4) | 158 (16.8) | 40 (4.2) | 550 (58.3) | 494 (52.4) |
| 2008 | 1,042 | 169 (16.2) | 263 (25.2) | 780 (74.9) | 629 (60.4) | 272 (26.1) | 4 (0.4) | 218 (20.9) | 50 (4.8) | 601 (57.7) | 540 (51.8) |
| 2009 | 1,100 | 168 (15.3) | 272 (24.7) | 836 (76.0) | 674 (61.3) | 293 (26.6) | 7 (0.6) | 231 (21.0) | 55 (5.0) | 639 (58.1) | 585 (53.2) |
| 2010 | 975 | 130 (13.3) | 282 (28.9) | 764 (78.4) | 636 (65.2) | 233 (23.9) | 6 (0.6) | 186 (19.1) | 41 (4.2) | 612 (62.8) | 561 (57.5) |
| 2011 | 1,048 | 164 (15.6) | 351 (33.5) | 788 (75.2) | 660 (63.0) | 240 (22.9) | 7 (0.7) | 192 (18.3) | 41 (3.9) | 644 (61.5) | 571 (54.5) |
| 2012 | 1,159 | 179 (15.4) | 371 (32.0) | 876 (75.6) | 722 (62.3) | 280 (24.2) | 5 (0.4) | 223 (19.2) | 52 (4.5) | 700 (60.4) | 623 (53.8) |
| 2013 | 1,256 | 208 (16.6) | 409 (32.6) | 932 (74.2) | 775 (61.7) | 292 (23.2) | 4 (0.3) | 229 (18.2) | 59 (4.7) | 756 (60.2) | 663 (52.8) |
| 2014 | 1,288 | 188 (14.6) | 409 (31.8) | 943 (73.2) | 846 (65.7) | 304 (23.6) | 11 (0.9) | 221 (17.2) | 72 (5.6) | 796 (61.8) | 700 (54.3) |
| 2015 | 1,224 | 209 (17.1) | 361 (29.5) | 878 (71.7) | 788 (64.4) | 263 (21.5) | 2 (0.2) | 191 (15.6) | 70 (5.7) | 752 (61.4) | 653 (53.3) |
| 2016 | 926 | 159 (17.2) | 238 (25.7) | 659 (71.2) | 557 (60.2) | 245 (26.5) | 5 (0.5) | 187 (20.2) | 53 (5.7) | 522 (56.4) | 454 (49.0) |
| 2017 | 893 | 186 (20.8) | 234 (26.2) | 594 (66.5) | 515 (57.7) | 236 (26.4) | 3 (0.3) | 174 (19.5) | 59 (6.6) | 471 (52.7) | 405 (45.4) |
| 2018 | 756 | 124 (16.4) | 166 (22.0) | 529 (70.0) | 453 (59.9) | 237 (31.3) | 4 (0.5) | 163 (21.6) | 70 (9.3) | 395 (52.2) | 354 (46.8) |
| 2019 | 787 | 146 (18.6) | 181 (23.0) | 562 (71.4) | 463 (58.8) | 205 (26.0) | 1 (0.1) | 163 (20.7) | 41 (5.2) | 436 (55.4) | 385 (48.9) |
| 2020 | 748 | 152 (20.3) | 129 (17.2) | 534 (71.4) | 404 (54.0) | 221 (29.5) | 1 (0.1) | 176 (23.5) | 44 (5.9) | 375 (50.1) | 343 (45.9) |
| 2021 | 813 | 176 (21.6) | 86 (10.6) | 567 (69.7) | 424 (52.2) | 254 (31.2) | 1 (0.1) | 202 (24.8) | 51 (6.3) | 383 (47.1) | 355 (43.7) |
| | | | | | | | | | | | |

© 2025 Cao C et al. *JAMA Network Open*.

| | | | | Any Treatment (%) | | | Single Treatment (%) | | | Multiple Treatments (%) | |
|---|---|---|---|---|---|---|---|---|---|---|---|
| | N | No Treatment *(%) | Surgery | Chemotherapy | Radiation | Only 1 Treatment | Surgery Only | Chemotherapy Only | Radiation Only | More than 1 Treatment | Chemoradiotherapy |
| Distant, Age<65y | | | | | | | | | | | |
| 2006 | 549 | 71 (12.9) | 188 (34.2) | 418 (76.1) | 377 (68.7) | 108 (19.7) | 3 (0.5) | 79 (14.4) | 26 (4.7) | 370 (67.4) | 320 (58.3) |
| 2007 | 607 | 102 (16.8) | 187 (30.8) | 470 (77.4) | 390 (64.3) | 108 (17.8) | 3 (0.5) | 90 (14.8) | 15 (2.5) | 397 (65.4) | 358 (59.0) |
| 2008 | 678 | 98 (14.5) | 185 (27.3) | 539 (79.5) | 426 (62.8) | 155 (22.9) | 2 (0.3) | 137 (20.2) | 16 (2.4) | 425 (62.7) | 387 (57.1) |
| 2009 | 725 | 88 (12.1) | 202 (27.9) | 587 (81.0) | 467 (64.4) | 182 (25.1) | 6 (0.8) | 148 (20.4) | 28 (3.9) | 455 (62.8) | 423 (58.3) |
| 2010 | 631 | 70 (11.1) | 207 (32.8) | 512 (81.1) | 428 (67.8) | 140 (22.2) | 3 (0.5) | 116 (18.4) | 21 (3.3) | 421 (66.7) | 382 (60.5) |
| 2011 | 676 | 74 (10.9) | 258 (38.2) | 548 (81.1) | 456 (67.5) | 146 (21.6) | 7 (1.0) | 120 (17.8) | 19 (2.8) | 456 (67.5) | 409 (60.5) |
| 2012 | 736 | 88 (12.0) | 265 (36.0) | 589 (80.0) | 478 (64.9) | 168 (22.8) | 4 (0.5) | 139 (18.9) | 25 (3.4) | 480 (65.2) | 423 (57.5) |
| 2013 | 799 | 104 (13.0) | 295 (36.9) | 636 (79.6) | 516 (64.6) | 174 (21.8) | 3 (0.4) | 146 (18.3) | 25 (3.1) | 521 (65.2) | 460 (57.6) |
| 2014 | 802 | 97 (12.1) | 295 (36.8) | 624 (77.8) | 548 (68.3) | 169 (21.1) | 6 (0.7) | 133 (16.6) | 30 (3.7) | 536 (66.8) | 473 (59.0) |
| 2015 | 746 | 108 (14.5) | 238 (31.9) | 580 (77.7) | 502 (67.3) | 139 (18.6) | 1 (0.1) | 113 (15.1) | 25 (3.4) | 499 (66.9) | 445 (59.7) |
| 2016 | 515 | 74 (14.4) | 148 (28.7) | 398 (77.3) | 324 (62.9) | 122 (23.7) | 2 (0.4) | 104 (20.2) | 16 (3.1) | 319 (61.9) | 283 (55.0) |
| 2017 | 490 | 88 (18.0) | 146 (29.8) | 351 (71.6) | 289 (59.0) | 122 (24.9) | 3 (0.6) | 101 (20.6) | 18 (3.7) | 280 (57.1) | 241 (49.2) |
| 2018 | 412 | 53 (12.9) | 99 (24.0) | 319 (77.4) | 260 (63.1) | 120 (29.1) | 2 (0.5) | 89 (21.6) | 29 (7.0) | 239 (58.0) | 222 (53.9) |
| 2019 | 366 | 44 (12.0) | 97 (26.5) | 299 (81.7) | 225 (61.5) | 97 (26.5) | 0 (0.0) | 88 (24.0) | 9 (2.5) | 225 (61.5) | 202 (55.2) |
| 2020 | 370 | 61 (16.5) | 75 (20.3) | 286 (77.3) | 225 (60.8) | 93 (25.1) | 0 (0.0) | 76 (20.5) | 17 (4.6) | 216 (58.4) | 202 (54.6) |
| 2021 | 410 | 69 (16.8) | 47 (11.5) | 314 (76.6) | 231 (56.3) | 123 (30.0) | 0 (0.0) | 106 (25.9) | 17 (4.1) | 218 (53.2) | 204 (49.8) |
| | | | | | | | | | | | |

© 2025 Cao C et al. *JAMA Network Open*.

| | | | | Any Treatment (%) | | | Single Treatment (%) | | | Multiple Treatments (%) | |
|---|---|---|---|---|---|---|---|---|---|---|---|
| | N | No Treatment *(%) | Surgery | Chemotherapy | Radiation | Only 1 Treatment | Surgery Only | Chemotherapy Only | Radiation Only | More than 1 Treatment | Chemoradiotherapy |
| Distant, Age≥65y | | | | | | | | | | | |
| 2006 | 287 | 78 (27.2) | 45 (15.7) | 170 (59.2) | 147 (51.2) | 83 (28.9) | 0 (0.0) | 59 (20.6) | 24 (8.4) | 126 (43.9) | 108 (37.6) |
| 2007 | 336 | 89 (26.5) | 69 (20.5) | 208 (61.9) | 174 (51.8) | 94 (28.0) | 1 (0.3) | 68 (20.2) | 25 (7.4) | 153 (45.5) | 136 (40.5) |
| 2008 | 364 | 71 (19.5) | 78 (21.4) | 241 (66.2) | 203 (55.8) | 117 (32.1) | 2 (0.5) | 81 (22.3) | 34 (9.3) | 176 (48.4) | 153 (42.0) |
| 2009 | 375 | 80 (21.3) | 70 (18.7) | 249 (66.4) | 207 (55.2) | 111 (29.6) | 1 (0.3) | 83 (22.1) | 27 (7.2) | 184 (49.1) | 162 (43.2) |
| 2010 | 344 | 60 (17.4) | 75 (21.8) | 252 (73.3) | 208 (60.5) | 93 (27.0) | 3 (0.9) | 70 (20.3) | 20 (5.8) | 191 (55.5) | 179 (52.0) |
| 2011 | 372 | 90 (24.2) | 93 (25.0) | 240 (64.5) | 204 (54.8) | 94 (25.3) | 0 (0.0) | 72 (19.4) | 22 (5.9) | 188 (50.5) | 162 (43.5) |
| 2012 | 423 | 91 (21.5) | 106 (25.1) | 287 (67.8) | 244 (57.7) | 112 (26.5) | 1 (0.2) | 84 (19.9) | 27 (6.4) | 220 (52.0) | 200 (47.3) |
| 2013 | 457 | 104 (22.8) | 114 (24.9) | 296 (64.8) | 259 (56.7) | 118 (25.8) | 1 (0.2) | 83 (18.2) | 34 (7.4) | 235 (51.4) | 203 (44.4) |
| 2014 | 486 | 91 (18.7) | 114 (23.5) | 319 (65.6) | 298 (61.3) | 135 (27.8) | 5 (1.0) | 88 (18.1) | 42 (8.6) | 260 (53.5) | 227 (46.7) |
| 2015 | 478 | 101 (21.1) | 123 (25.7) | 298 (62.3) | 286 (59.8) | 124 (25.9) | 1 (0.2) | 78 (16.3) | 45 (9.4) | 253 (52.9) | 208 (43.5) |
| 2016 | 411 | 85 (20.7) | 90 (21.9) | 261 (63.5) | 233 (56.7) | 123 (29.9) | 3 (0.7) | 83 (20.2) | 37 (9.0) | 203 (49.4) | 171 (41.6) |
| 2017 | 403 | 98 (24.3) | 88 (21.8) | 243 (60.3) | 226 (56.1) | 114 (28.3) | 0 (0.0) | 73 (18.1) | 41 (10.2) | 191 (47.4) | 164 (40.7) |
| 2018 | 344 | 71 (20.6) | 67 (19.5) | 210 (61.0) | 193 (56.1) | 117 (34.0) | 2 (0.6) | 74 (21.5) | 41 (11.9) | 156 (45.3) | 132 (38.4) |
| 2019 | 421 | 102 (24.2) | 84 (20.0) | 263 (62.5) | 238 (56.5) | 108 (25.7) | 1 (0.2) | 75 (17.8) | 32 (7.6) | 211 (50.1) | 183 (43.5) |
| 2020 | 378 | 91 (24.1) | 54 (14.3) | 248 (65.6) | 179 (47.4) | 128 (33.9) | 1 (0.3) | 100 (26.5) | 27 (7.1) | 159 (42.1) | 141 (37.3) |
| 2021 | 403 | 107 (26.6) | 39 (9.7) | 253 (62.8) | 193 (47.9) | 131 (32.5) | 1 (0.2) | 96 (23.8) | 34 (8.4) | 165 (40.9) | 151 (37.5) |
| | | | | | | | | | | | |

© 2025 Cao C et al. *JAMA Network Open*.

| | | | | Any Treatment (%) | | | Single Treatment (%) | | | Multiple Treatments (%) | |
|---|---|---|---|---|---|---|---|---|---|---|---|
| | N | No Treatment *(%) | Surgery | Chemotherapy | Radiation | Only 1 Treatment | Surgery Only | Chemotherapy Only | Radiation Only | More than 1 Treatment | Chemoradiotherapy |
| Distant, Metropolitan | | | | | | | | | | | |
| 2006 | 710 | 121 (17.0) | 191 (26.9) | 508 (71.5) | 450 (63.4) | 164 (23.1) | 3 (0.4) | 118 (16.6) | 43 (6.1) | 425 (59.9) | 372 (52.4) |
| 2007 | 798 | 155 (19.4) | 212 (26.6) | 583 (73.1) | 483 (60.5) | 175 (21.9) | 1 (0.1) | 140 (17.5) | 34 (4.3) | 468 (58.6) | 424 (53.1) |
| 2008 | 906 | 147 (16.2) | 227 (25.1) | 680 (75.1) | 546 (60.3) | 239 (26.4) | 3 (0.3) | 192 (21.2) | 44 (4.9) | 520 (57.4) | 470 (51.9) |
| 2009 | 954 | 148 (15.5) | 228 (23.9) | 721 (75.6) | 587 (61.5) | 255 (26.7) | 6 (0.6) | 200 (21.0) | 49 (5.1) | 551 (57.8) | 508 (53.2) |
| 2010 | 833 | 107 (12.8) | 239 (28.7) | 658 (79.0) | 549 (65.9) | 199 (23.9) | 4 (0.5) | 161 (19.3) | 34 (4.1) | 527 (63.3) | 485 (58.2) |
| 2011 | 901 | 129 (14.3) | 302 (33.5) | 686 (76.1) | 571 (63.4) | 214 (23.8) | 7 (0.8) | 172 (19.1) | 35 (3.9) | 558 (61.9) | 492 (54.6) |
| 2012 | 983 | 143 (14.5) | 314 (31.9) | 753 (76.6) | 615 (62.6) | 243 (24.7) | 5 (0.5) | 196 (19.9) | 42 (4.3) | 597 (60.7) | 533 (54.2) |
| 2013 | 1,081 | 176 (16.3) | 352 (32.6) | 806 (74.6) | 661 (61.1) | 250 (23.1) | 3 (0.3) | 201 (18.6) | 46 (4.3) | 655 (60.6) | 565 (52.3) |
| 2014 | 1,102 | 163 (14.8) | 347 (31.5) | 806 (73.1) | 725 (65.8) | 255 (23.1) | 9 (0.8) | 190 (17.2) | 56 (5.1) | 684 (62.1) | 601 (54.5) |
| 2015 | 1,049 | 177 (16.9) | 306 (29.2) | 746 (71.1) | 679 (64.7) | 224 (21.4) | 2 (0.2) | 161 (15.3) | 61 (5.8) | 648 (61.8) | 555 (52.9) |
| 2016 | 785 | 132 (16.8) | 204 (26.0) | 566 (72.1) | 472 (60.1) | 207 (26.4) | 3 (0.4) | 161 (20.5) | 43 (5.5) | 446 (56.8) | 388 (49.4) |
| 2017 | 753 | 152 (20.2) | 192 (25.5) | 505 (67.1) | 431 (57.2) | 208 (27.6) | 3 (0.4) | 153 (20.3) | 52 (6.9) | 393 (52.2) | 338 (44.9) |
| 2018 | 646 | 108 (16.7) | 138 (21.4) | 446 (69.0) | 385 (59.6) | 203 (31.4) | 4 (0.6) | 138 (21.4) | 61 (9.4) | 335 (51.9) | 297 (46.0) |
| 2019 | 685 | 125 (18.2) | 156 (22.8) | 488 (71.2) | 410 (59.9) | 178 (26.0) | 1 (0.1) | 137 (20.0) | 40 (5.8) | 382 (55.8) | 339 (49.5) |
| 2020 | 629 | 129 (20.5) | 109 (17.3) | 445 (70.7) | 345 (54.8) | 182 (28.9) | 1 (0.2) | 142 (22.6) | 39 (6.2) | 318 (50.6) | 291 (46.3) |
| 2021 | 674 | 147 (21.8) | 69 (10.2) | 472 (70.0) | 353 (52.4) | 206 (30.6) | 0 (0.0) | 166 (24.6) | 40 (5.9) | 321 (47.6) | 298 (44.2) |
| | | | | | | | | | | | |

© 2025 Cao C et al. *JAMA Network Open*.

| | | | | Any Treatment (%) | | | Single Treatment (%) | | | Multiple Treatments (%) | |
|---|---|---|---|---|---|---|---|---|---|---|---|
| | N | No Treatment *(%) | Surgery | Chemotherapy | Radiation | Only 1 Treatment | Surgery Only | Chemotherapy Only | Radiation Only | More than 1 Treatment | Chemoradiotherapy |
| Distant, Nonmetropolitan | | | | | | | | | | | |
| 2006 | 126 | 28 (22.2) | 42 (33.3) | 80 (63.5) | 74 (58.7) | 27 (21.4) | 0 (0.0) | 20 (15.9) | 7 (5.6) | 71 (56.3) | 56 (44.4) |
| 2007 | 144 | 36 (25.0) | 44 (30.6) | 94 (65.3) | 80 (55.6) | 27 (18.8) | 3 (2.1) | 18 (12.5) | 6 (4.2) | 81 (56.3) | 69 (47.9) |
| 2008 | 136 | 22 (16.2) | 36 (26.5) | 100 (73.5) | 83 (61.0) | 33 (24.3) | 1 (0.7) | 26 (19.1) | 6 (4.4) | 81 (59.6) | 70 (51.5) |
| 2009 | 146 | 20 (13.7) | 44 (30.1) | 115 (78.8) | 87 (59.6) | 38 (26.0) | 1 (0.7) | 31 (21.2) | 6 (4.1) | 88 (60.3) | 77 (52.7) |
| 2010 | 141 | 23 (16.3) | 43 (30.5) | 105 (74.5) | 87 (61.7) | 33 (23.4) | 2 (1.4) | 24 (17.0) | 7 (5.0) | 85 (60.3) | 76 (53.9) |
| 2011 | 145 | 35 (24.1) | 49 (33.8) | 101 (69.7) | 87 (60.0) | 25 (17.2) | 0 (0.0) | 20 (13.8) | 5 (3.4) | 85 (58.6) | 78 (53.8) |
| 2012 | 175 | 36 (20.6) | 57 (32.6) | 122 (69.7) | 107 (61.1) | 36 (20.6) | 0 (0.0) | 26 (14.9) | 10 (5.7) | 103 (58.9) | 90 (51.4) |
| 2013 | 172 | 31 (18.0) | 56 (32.6) | 124 (72.1) | 112 (65.1) | 42 (24.4) | 1 (0.6) | 28 (16.3) | 13 (7.6) | 99 (57.6) | 96 (55.8) |
| 2014 | 185 | 25 (13.5) | 61 (33.0) | 136 (73.5) | 120 (64.9) | 49 (26.5) | 2 (1.1) | 31 (16.8) | 16 (8.6) | 111 (60.0) | 98 (53.0) |
| 2015 | 172 | 32 (18.6) | 55 (32.0) | 130 (75.6) | 106 (61.6) | 38 (22.1) | 0 (0.0) | 30 (17.4) | 8 (4.7) | 102 (59.3) | 96 (55.8) |
| 2016 | 141 | 27 (19.1) | 34 (24.1) | 93 (66.0) | 85 (60.3) | 38 (27.0) | 2 (1.4) | 26 (18.4) | 10 (7.1) | 76 (53.9) | 66 (46.8) |
| 2017 | 138 | 32 (23.2) | 42 (30.4) | 89 (64.5) | 84 (60.9) | 28 (20.3) | 0 (0.0) | 21 (15.2) | 7 (5.1) | 78 (56.5) | 67 (48.6) |
| 2018 | 109 | 16 (14.7) | 28 (25.7) | 83 (76.1) | 67 (61.5) | 33 (30.3) | 0 (0.0) | 25 (22.9) | 8 (7.3) | 60 (55.0) | 57 (52.3) |
| 2019 | 102 | 21 (20.6) | 25 (24.5) | 74 (72.5) | 53 (52.0) | 27 (26.5) | 0 (0.0) | 26 (25.5) | 1 (1.0) | 54 (52.9) | 46 (45.1) |
| 2020 | 117 | 22 (18.8) | 20 (17.1) | 88 (75.2) | 58 (49.6) | 39 (33.3) | 0 (0.0) | 34 (29.1) | 5 (4.3) | 56 (47.9) | 51 (43.6) |
| 2021 | 137 | 28 (20.4) | 17 (12.4) | 94 (68.6) | 70 (51.1) | 48 (35.0) | 1 (0.7) | 36 (26.3) | 11 (8.0) | 61 (44.5) | 56 (40.9) |

*including unknown. US, United States; y, years.

© 2025 Cao C et al. *JAMA Network Open*.
