# Supplementary material for: Updated Estimates of Patients With Oropharyngeal Cancer in the US
Source: JAMA Netw Open. 2025 Oct 24;8(10):e2539258. doi: 10.1001/jamanetworkopen.2025.39258 (PMC12552931; doi:10.1001/jamanetworkopen.2025.39258)
Supplement: Supplement 2. — Data Sharing Statement [file jamanetwopen-e2539258-s002.pdf]

Data Sharing Statement

Cao. Updated Estimates of Patients With Oropharyngeal Cancer in the US. *JAMA Netw Open*. Published October 24, 2025. doi:10.1001/jamanetworkopen.2025.39258

Data
Data available: Yes
Data types: Other (please specify)
Additional Information: http://seer.cancer.gov
How to access data: http://seer.cancer.gov
When available: With publication

Supporting Documents
Document types: None

Additional Information
Who can access the data: Not applicable.
Types of analyses: Not applicable.
Mechanisms of data availability: Not applicable.
